# The Role of Kupffer Cells and Liver Macrophages in the Pathogenesis of Metabolic Dysfunction-Associated Steatotic Liver Disease

**DOI:** 10.3390/biomedicines14010151

**Published:** 2026-01-11

**Authors:** Ioannis Tsomidis, Angeliki Tsakou, Argyro Voumvouraki, Elias Kouroumalis

**Affiliations:** 1 Laboratory of Gastroenterology and Hepatology, University of Crete School of Medicine, Voutes Campus, 70013 Heraklion, Crete, Greece; itsomidi@gmail.com; 2 1st Department of Internal Medicine, AHEPA University Hospital, 54621 Thessaloniki, Greece; angtsak207@gmail.com (A.T.); iro_voum@yahoo.gr (A.V.); 3 Department of Gastroenterology, PAGNI University Hospital, University of Crete School of Medicine, 71500 Heraklion, Crete, Greece

**Keywords:** NAFLD, MASLD, MASH, pathogenesis, Kupffer cells, bone-marrow derived macrophages

## Abstract

Metabolic dysfunction-associated steatotic liver disease (MASLD) is a continuum of hepatic pathological manifestations of the metabolic syndrome. Pathogenesis is not clearly understood despite recent progress, but Kupffer cells and bone marrow-derived macrophages (BMDMs) have a fundamental role. In this review, the multiple pathophysiological aspects of MASLD are presented, including genetics, insulin resistance, lipotoxicity, and inflammation. The participation of innate and adaptive immunity, as well as the implications of the recently described trained immunity, is presented. The interplay of the liver with the gut microbiota is also analyzed. A recent adipocentric theory and the various mechanisms of hepatocyte death are also described. The fundamental role of Kupffer cells and other liver macrophages is discussed in detail, including their extreme phenotypic plasticity in both the normal and the MASLD liver. The functional differentiation between pro-inflammatory and anti-inflammatory subpopulations and their protective or detrimental involvement is further described, including the participation of Kupffer cells and BMDMs in all aspects of MASLD pathogenesis. The role of macrophages in the development of advanced MASLD, including fibrosis and hepatocellular carcinoma, is analyzed and the lack of explanation for the transition from MASLD to MASH is recognized. Finally, current modalities of drug treatment are briefly presented and the effects of different drugs on macrophage polarization and functions are discussed.

## 1. Introduction

MASLD is characterized by fat deposition in more than 5% of hepatocytes without another identifiable cause for secondary fat deposition, such as alcohol consumption. MASLD may progress to hepatitis (MASH), fibrosis, cirrhosis, or hepatocellular carcinoma (HCC) (8). MASLD is the hepatic manifestation of metabolic syndrome, with a strong genetic background, and is associated with metabolic co-morbidities, including obesity, diabetes melitus, dyslipidaemia, and hypertension [1,2,3]. It is the most common cause of chronic liver disease globally, affecting up to 25–38% of the general adult population, with considerable clinical and economic burden [4,5]. There seems to be a sex difference, as men more commonly proceed to cirrhosis than women [6].

It was described for the first time by Ludwig under the name non-alcoholic fatty liver disease (NAFLD) and non-alcoholic steatohepatitis (NASH) [7]. Metabolic dysfunction-associated fatty liver disease (MAFLD) was proposed as a new name in 2020 [8] and metabolic dysfunction-associated steatotic liver disease (MASLD) appeared in 2023 in an effort to replace NAFLD. The new names were based on non-stigmatizing diagnostic criteria and aimed to improve patient awareness [9,10]. We feel that NAFLD is a better term and pays tribute to Ludwig for his excellent description of the disease. The argument that a disease should be defined by a positive instead of a negative definition is superficial, as the use of alcohol should be excluded in any definition. However, the terms MASLD and MASH will be used in this review to avoid confusion, since they are used in most recent papers. It should be stressed, however, that in terms of diagnosis, no real difference exists regardless of whatever name is used, as proven by comparisons of different epidemiology reports [11].

The liver is the central organ affected by the disease, with the cardiometabolic co-morbidities comprising physiopathological satellites, despite the fact that these satellites are the main etiology of mortality and morbidity. Moreover, the multifactorial pathogenetic aspects of this condition remain only partially elucidated.

A crucial and as yet unexplained aspect of MASLD is that not all patients with simple steatosis will progress to MASH [12]. Additionally, deposition of fat in adipose tissue or the liver may, in fact, protect from systemic cardiovascular disease. This group of patients is named healthy obese individuals [13]. Furthermore, a significant number of patients with morbid obesity have normal liver histology, reflecting the complex pathophysiology of these conditions [14]. While the reasons for these different behaviors remain unclear, it is plausible that the activation of hepatic macrophages may be the decisive factor in the progression of MALSD, as activated macrophages in turn activate hepatic stellate cells (HSCs), leading to their trans-differentiation into collagen-producing myofibroblasts, which in turn drive the fibrosis characteristic of MASH [15]. Liver macrophages have a critical role in each and every step of the pathologic development of MASLD. At least four types of liver macrophages reside in the liver. Kupffer cells reside in the sinusoids, where they can arrest and phagocytose blood-borne pathogens. Liver capsule macrophages reside beneath the mesothelium on the liver surface. Central vein macrophages are localized near the central vein, while bile duct macrophages are located near epithelial bile ducts. Expression of different genes and proteins is used for their distinction [16].

Kupffer cells (KCs) are tissue-resident macrophages that infiltrate the liver early during embryogenesis. Soon after liver colonization, KCs acquire a tissue-specific characteristic and mature in parallel with the developing liver. During liver development and later in life, KCs functions are critical for liver homeostasis, including erythrocyte and iron recycling and liver metabolism [17,18]. In addition to clearance of aged RBCs and platelets, KCs also have important anti-tumor functions, such as coordination of tumoricidal immune responses [19] and phagocytosis of circulating tumor cells [20]. The exact role of macrophages in the regulation of MASLD progression remains unclear and will be discussed in this review. First, an overview of MASLD pathogenetic pathways will be presented, followed by the involvement of liver macrophages in the same pathways.

## 2. An Overview of MASLD/MASH Pathogenesis

Over two decades ago, the ‘two-hit’ theory of MASLD pathogenesis was proposed [21]. The first hit is fat deposition in the hepatocytes, which sensitizes the liver for the second hit inflicted by factors such as oxidative stress, endotoxins, lipotoxicity, and necroinflammation. Steatosis of hepatocytes increases insulin resistance, endoplasmic reticulum (ER) stress, and abnormalities of autophagy, collectively known as lipotoxicity. Immune reactions in KCs promote tissue infiltration by neutrophils and lymphocytes, ultimately leading to hepatocyte death and fibrosis [22]. This hypothesis has been gradually revised as new theories emerge. The “multiple parallel hits” pathogenetic hypothesis has replaced the two-hit theory [23]. This hypothesis considers MASLD pathogenesis as a complex condition implicating crosstalk between metabolically active tissues such as adipose tissue and the liver/gut axis. Multiple abnormalities, including insulin resistance, hepatic steatosis, oxidative stress, mitochondrial dysfunction, abnormal gut microbiota, and genetic factors, may run in parallel [23].

But if this is the case, and multiple insults act in parallel, the fact that almost 70% of patients with fatty liver do not progress to hepatitis or cirrhosis remains unexplained. Moreover, the relative contribution of every single insult remains to be clarified. It is therefore plausible that a somewhat modified “two hit” theory may better suit the pathogenesis of MASLD. Insulin resistance with or without obesity may be the initial event leading to lipid overload of hepatocytes. The three main sources of fatty acids in the liver include hepatic de novo lipogenesis, free fatty acids from adipose tissue, and the uptake of dietary fats [24]. The content of hepatic fatty acids derived from de novo lipogenesis contributes about 26%, in contrast to 5% in normal healthy individuals [24]. The excessive accumulation of triglycerides in the liver is mainly due to the increased availability of free fatty acids through lipolysis in insulin-resistant and hypertrophied adipose tissue. In addition, increased fatty acid synthesis leads to decreased mitochondrial fatty acid oxidation. Sterol element binding protein 1c (SREBP1c) is the main transcription factor involved in regulating hepatic de novo lipogenesis. This transcription factor is synthesized as an inactive precursor, and its proteolytic maturation is initiated in the membrane of the endoplasmic reticulum upon stimulation by insulin. SREBP cleavage-activating protein (SCAP) is required as a chaperon protein to escort SREBP from the endoplasmic reticulum and to facilitate the proteolytic release of the N-terminal domain of SREBP into the Golgi. SCAP inhibition prevents activation of SREBP and inhibits the expression of genes involved in triglyceride and fatty acid synthesis, resulting in the inhibition of de novo lipogenesis [25]. SREBP1c is a critical modulator involved in the regulation of hepatic de novo lipogenesis by insulin through preferential activation of genes involved in fatty acid synthesis, such as fatty acid synthase, whereas SREBP2 preferentially activates genes required for cholesterol synthesis [26]. SREBP1c binds to the promoter of the patatin-like phospholipase domain containing protein 3 (PNPLA3) and regulates its expression. This enzyme acts as a triacylglycerol lipase that mediates the hydrolysis of triacylglycerol in adipocytes, and thus, hepatic lipid accumulation is stimulated by the accumulation of PNPLA3 in the lipid droplets of hepatocytes, promoting hepatic fat accumulation [27].

The hepatocyte accumulation of free fatty acids (FFAs) initiates mitochondrial dysfunction and excessive reactive oxygen species (ROS) production, leading to lipotoxic intermediates and mitochondrial DNA damage. Lipotoxicity further exacerbates hepatocyte damage [28]. ER stress constitutes a potential “second hit” that causes the secretion of inflammatory and fibrogenic cytokines such as IL-6, TNFα, IL-1β, and TGFβ1 [29] followed by a cascade of events that exacerbate the inflammation and development of fibrosis. Such a model can accommodate both the parallel action of insults and also a sequential series of events. ER stress is associated with changes in the gut microbiota, alterations in gut permeability, entrance of bacterial products such as bacterial lipopolysacharide (LPS) into the portal blood, and activation of toll-like receptors (TLRs) in KCs. Activated KCs promote the recruitment of monocyte-derived macrophages that further damage the liver [30]. On the other hand, adipose tissue contributes to insulin resistance by secreting adipokines such as leptin and adiponectin. Damage-associated molecular patterns (DAMPs) from injured hepatocytes and gut-derived pathogen-associated molecular patterns (PAMPs) synergize to activate innate immunity and amplify cytokine-mediated inflammation. KC activation further increases ROS production, while ROS-induced pathways maintain inflammatory gene expression in a vicious circle [31].

Several additional factors, such as genetics, dietary habits, air pollution, and smoking, are implicated in the initiation of MASLD [32,33,34]. Recently, oral conditions, such as periodontitis, have been associated with the development of MASLD [35,36]. The factors that are implicated in the pathogenesis of MASLD will be further analyzed.

### 2.1. Genetics

A 35–61% heritable component is implicated in MASLD and MASH [37]. Although MASLD is strongly associated with obesity, approximately 20% of patients with hepatic fat deposition have a normal body mass index, a condition reported as lean MASLD, when they have one or more cardiac risk factors and cryptogenic steatotic liver disease, when no cardiac risk factors exist. The discovery of a single-nucleotide polymorphism (SNP) in the PNPLA3 gene disclosed a role of this enzyme in regulating hepatocyte lipid metabolism [38]. This SNP is associated with enhanced MASLD risk and is resistant to ubiquitation and degradation, leading to abnormal triglyceride mobilization and deposition in the hepatocyte [39]. This SNP is also associated with a high risk of fibrosis through its effect on HSCs. In these cells, wild-type PNPLA3 hydrolyzes retinyl esters to enhance extracellular retinol release, which is reduced when the SNP is present, leading to increased trans-differentiation of stellate cells into myofibroblasts [40]. The PNPLA3 SNP is also associated with a 12-fold greater risk of HCC in patients with MALSD and the homozygous variant compared with a healthy population [41]. Risks associated with the PNPLA3 SNP have been confirmed in several independent cohorts [42]. Current research indicates that the PNPLA3 rs738409 variant is more common among individuals with lean MASLD than their overweight or obese counterparts. Interestingly, data also suggest that individuals carrying the PNPLA3 GG genotype seem to have a greater reduction in fat deposition and liver enzymes after lifestyle intervention and drug treatment of MASLD [43].

Additional genes implicated in MASLD have been identified in genome-wide association studies (GWAS) including TM6SF2 (transmembrane 6 superfamily member 2), MBOAT7 (membrane bound O acyltransferase 7), and GCKR (glucokinase regulator) [44].

The association of PNPLA3, TM6SF2, GCKR, MBOAT7, and the protective HSD17B13 SNPs has been repeatedly confirmed with MASLD progression. These genetic variants play important roles in the secretion of hepatic very low-density lipoprotein and lipogenesis [45].

Identification of different genetic SNPs has been reported in non-European populations. A GWAS study indicated that 11 SNPs of PNPLA3 and SAMM50 genes are associated with the development of MASLD in a Taiwanese population [46]. In a Brazilian study, TM6SF2 rs58542926, GCKR rs1260326 and rs780094, and HSD17B13 rs72613567 were not associated with liver fibrosis. On the contrary, the PNPLA3 GG genotype was more frequent in F2-F4 (23%) and F0-F1 (22%) patients with fibrosis compared to controls (9%). The MBOAT7 TT genotype was significantly related to fibrosis, with a prevalence of 23% in F2-F4 patients compared to 10% in F0-F1 and 11% in controls. The protective MTARC1 AA genotypes were more frequent in controls (52%) when compared to patients with fibrosis (5% *p* = 2.76 × 10^−20^) [47]. An Egyptian study found that protein tyrosine phosphatase non-receptor type 2 (PTPN2, rs2542151) rs2542151 and MBOAT7 rs641738 SNPs were associated with MASLD susceptibility, but only the PTPN2 rs2542151 mutation was related to fibrosis progression [48].

Apart from GWAS, transcriptome association studies (TWAS) are able to identify significant new associated genes in MASLD and will possibly widen the related field [49].

### 2.2. Insulin Resistance and Lipotoxicity

Insulin resistance (IR) and lipotoxicity are initial events in MASLD. Insulin resistance development has three pillars, namely the liver, the skeletal muscles, and the white adipose tissue (WAT). The liver is a major source of endogenous glucose production, which is inhibited by insulin. In obesity this inhibitory effect is diminished, while the stimulatory effect of insulin on lipogenesis is not affected, resulting in liver insulin resistance, hyperglycemia, hyperinsulinemia, and hepatic fat deposition [50,51]. Obesity also induces systemic and local inflammation, promoting the development of steatosis. Pro-inflammatory cytokines induce lipogenic gene expression and promote de novo lipogenesis [52]. The same cytokines induce genes implicated in ceramide biosynthesis, leading to increased intrahepatic ceramide levels, which impair insulin signaling [53].

Consumption of a high-energy Western diet rich in sucrose, fructose, and saturated fat results in MASLD induction and increased ectopic fat accumulation in skeletal muscle. Increased fat in myocytes appears prior to onset of MASLD and causes muscle insulin resistance, leading to decreased insulin-stimulated glucose transport and muscle glycogen synthesis. Storage of ingested glucose as muscle glycogen is not efficient. Therefore, it is redirected to the liver, where it stimulates SREBP1c, which regulates de novo lipid synthesis (DNL), leading to increased VLDL production, hypertriglyceridemia, and MASLD. Monosaccharides can also recruit other transcription factors such as PPARγ coactivator 1-β, and LXR to further increase hepatocyte lipogenesis.

WAT insulin resistance and inflammation enhance lipolysis and increase fatty acid delivery to the liver, leading to increased fatty acid esterification into hepatic triglycerides and MASLD. Triglyceride-rich lipoproteins (TRLs) are metabolized in the circulation by peripheral lipoprotein lipase (Lpl). Endogenous Lpl inhibitors reduce peripheral triglyceride metabolism, increasing hepatic triglyceride uptake from TRLs, leading to increased fat deposition in the liver and skeletal muscle [54].

All types of fat and sugars do not influence fat deposition the same way, even when diets are isocaloric. Different metabolic pathways mediate the effects of carbohydrates and fat intake on liver steatosis. Fat acts through dysregulation of lipid storage or through increased lipolysis, whereas carbohydrates enhance liver fat accumulation through de novo lipogenesis. Saturated fat and fructose induce the greatest increase in intrahepatic triglycerides, insulin resistance, and harmful ceramides compared with unsaturated fats, which in fact seem to be protective [55].

De novo lipid biosynthesis occurs after consumption of excessive carbohydrates or when circulating insulin levels are high. Carbohydrates generate acetyl-CoA after glycolysis, which serves as a substrate for fatty acid and cholesterol synthesis. The degree of hepatic steatosis varies in lean and healthy obese individuals depending on the food composition, age, and use of medications [56,57].

The reason for the progression from MAFLD to MASH is virtually unknown, as mentioned before. However, the mechanism for the transition has been better clarified. In addition to the initiation of hepatic insulin resistance, lipotoxicity is involved in the progression of MASLD. Accumulation of toxic lipids induce cellular stress and ER stress and trigger the unfolded protein response (UPR), which is an adaptive cell-protective stress response. In MASLD, chronicity of UPR activation aggravates fat deposition and insulin resistance, leading to inflammation, oxidative stress, and hepatocellular death [58]. Mitochondrial dysfunction and reduced hepatocyte capacity to oxidize excess lipids further aggravate lipotoxicity and lead to elevated production of reactive oxygen species (ROS), resulting in more oxidative stress, inflammation, and fibrosis [59,60], thus introducing a vicious circle. Ultimately, death of the hepatocytes occurs through multiple cellular pathways [59,61].

A particular note should be made for lipotixicity mediated by hepatic free cholesterol overload, which drives necro-inflammation and fibrosis in several animal models with MASH. Free cholesterol in hepatocytes can cause ER stress, mitochondrial damage, induction of toxic oxysterols, and cholesterol crystallization in lipid droplets, leading to hepatocyte apoptosis, necrosis, or pyroptosis [62].

On the other hand, there are factors to protect liver from oxidative stress. Defense mechanisms to protect from excess ROS have been identified. Nuclear factor erythroid 2-related factor2 (Nrf2) is an important element in this defense mechanism. Nrf2 accurately monitors intracellular redox status. It is bound to Kelch-like ECH-associated protein1 (Keap1) in the cytoplasm from which it is detached when ROS levels are increased and is transported to the nucleus, where it increases the transcription of protective genes [63]. Nrf2 is also another protector against hepatic lipid stress. Nrf1 delays MASLD progression, but Nrf2 attenuates MASH. In combination, they act synergistically against steatosis and may even facilitate liver repair [64,65].

Detailed reviews of lipid abnormalities in MASLD have been recently published [54,66,67].

### 2.3. Inflammation

Inflammation in MASLD is a critical turning point that possibly separates steatosis from steatohepatitis and cirrhosis. It is regulated by several intrahepatic and extrahepatic factors [68] derived from the gut, adipose tissue, skeletal muscle, and bone marrow [69,70,71,72,73].

Triggers of inflammation in MASLD include a hypercaloric diet, obesity, and lifestyle. Stressed hepatocytes release pro-inflammatory mediators and DAMPs, leading to liver immune cell activation and infiltration by bone marrow-derived immune cells, further damaging hepatocytes. Hepatocyte death occurs as well as hepatocyte senescence, increasing the immune response. Liver inflammation is also enhanced by several extrahepatic systems, including the adipose tissue, gut, and skeletal muscle, as mentioned before [74]. Inflammation is tightly connected with the immunological response.

### 2.4. Traditional Innate Immunity

Hepatocyte damage, inflammation, and apoptosis caused by toxic lipids lead to the liberation of DAMPs that activate innate immune cells and promote inflammation by inducing NF-κB and NLRP3 inflammasome-related pathways. This condition also impairs the mitochondrial function, causing the release of mitochondrial DNA, which together with other DAMPs is sensed by hepatic macrophages and promotes the secretion of pro-inflammatory cytokines IL-1β and IL-18 [75,76] and chemokines that propagate tissue injury and recruit adaptive immune effectors [77,78]. Bone marrow-derived macrophages (BMDMs) are recruited to the inflamed liver via chemokines such as CCL2. MASH may also promote adipose tissue inflammation, further enhancing MASH [79].

### 2.5. Adaptive Immune Cells in MASH Progression

Several subsets of T cells (CD4+, CD8+, γδ, and Treg) and B cells are participating in MASLD and its progression to MASH indicating the significance of adaptive immunity [80,81,82].

CD4+ T cells are characterized by plasticity, participating in either pro- or anti-inflammatory responses via distinct subsets such as Th1, Th2, Th17, and T regulatory (Treg) cells [83,84]. In MASLD, there is evidence of a Th1/Th17 shift and increased production of IFNγ and IL-17A, enhancing inflammation and fibrosis [85,86,87]. Inhibition of liver recruitment of these cells ameliorates liver damage in mice, indicating their role in pathogenesis [88].

The relative number of CD8+ cells is elevated in the livers of MASH patients [89] and amplifies liver injury through IFNγ/TNF release and cytotoxicity. However, mitochondrial dysfunction, which is common in MASH, impairs CD8+ T-cell mobility and tumor responses [90], thus facilitating HCC development. On the other hand, they can participate in the resolution of inflammation during regression [90,91,92].

The role of B cells has not been well clarified in MASLD progression. There is evidence that they contribute to MASH development by producing pro-inflammatory cytokines (IL-6, TNFa) and by influencing a CD4+ T-cell shift toward Th1/Th17 phenotypes [93,94]. Additionally, a loss of IL-10-producing regulatory B cells has been reported in MASLD models [95]. Interestingly, IgA+ B cells have been involved in the modulation of BMDM polarization and promotion of fibrogenesis [96].

In summary, data indicate that complex crosstalk between innate and adaptive immune cells regulates the liver inflammatory environment that is characteristic of MASH [97]. Initially, innate immune cells such as KCs, neutrophils, and dendritic cells respond to lipotoxic elements and gut-derived microbial products by producing pro-inflammatory cytokines and chemokines that recruit and activate adaptive immune populations [98]. On the other hand, CD4+ and CD8+ T cells participate in the damage of hepatocytes through cytotoxic mechanisms and the secretion of IFNγ and TNF-α [98]. Moreover, unconventional T cells such as Mucosal-Associated Invariant T cells (MAIT) and γδ T cells may either exacerbate inflammation or promote disease regression, depending on the metabolic and cytokine environment [96]. It is mechanistically accepted that this interplay drives the transition from MASLD to MASH. In this interplay, immune cells of the adipose tissue also participate in regulating lipolysis and insulin action [99,100,101,102] as mentioned above. Chronic metabolic inflammation, also known as “metaflammation” and immunometabolism in MASLD, has been recently reviewed [103,104].

### 2.6. Trained Immunity

The mechanisms described so far constitute the “traditional” immunity (TI) contribution to MASLD progression. However, the introduction of this so-called trained immunity has modified the classical distinction between innate and adaptive immunity. Indeed, innate immune cells can undergo long-term functional reprogramming [105] which is initiated by either exogenous antigenic stimuli such as microbial components or by endogenous danger signals, including oxidized lipids, uric acid, and the heme group [105,106]. These stimuli induce a strong and nonspecific inflammatory response upon subsequent challenges by the same antigens. Trained immunity and tolerance are two opposite functional programs of innate immunity. However, dysregulation of trained immunity can lead to a persistent state of immunological tolerance, a mechanism that downregulates the inflammatory response, contributing to disease progression [105]. Unlike adaptive immunity, trained immunity is not dependent on antigen specificity or clonal expansion, yet it can persist for protracted periods of time [107,108].

In MASLD, the implications of TI appear mostly harmful [109]. In this model, immunological memory also applies to the innate immune arm in addition to the adaptive arm [108]. These rewired cells exhibit a strong and protracted pro-inflammatory and pro-fibrotic profile, with increased production of cytokines, chemokines, and matrix remodeling enzymes [109] leading to the perpetuation of hepatic inflammation and progression from simple steatosis to hepatitis, fibrosis, and HCC [77,109].

Another advance in immunology that has allowed for further dissection of individual immune components and their respective roles in MASLD pathology is the recent description of the bidirectional communication between innate and adaptive immunity. The contribution of such communications to MASLD is not yet fully clarified [98].

In summary, the interplay between “Traditional” and “Trained” immunity pathways participates in MASLD to MASH progression. In the early stages, hepatocyte lipid deposition and oxidative stress activate traditional innate immunity, assisted by adaptive immunity. Protracted metabolic and microbial stimuli induce trained immunity, characterized by reprogramming of innate immune cells. These rewired cells maintain hepatic injury, driving progression from steatosis to steatohepatitis.

Details can be found in recently published reviews [108,109,110].

### 2.7. Gut Microbiota and MASLD

Traditional theories have focused on insulin resistance, lipotoxicity, oxidative stress, and chronic inflammation as the main pathogenetic factors in MASLD. These factors are no longer sufficient to explain the variability of the disease [77,111,112].

One of the factors that has gained attention in recent years is the intestinal microbiome. Intestinal dysbiosis is strongly associated with MASLD and MASH [113,114]. Interestingly, alterations of the intestinal virome and mycobiome were also implicated in the pathogenesis of MASH [115,116]. Several factors affect gut dysbiosis, including the environment, dietary habits, and medications. Liver-related factors, such as inflammation, and gut-related factors, such as dysfunction of the intestinal barrier, are also implicated. In a murine model with MASLD, early increased lipid deposition in the hepatocytes can cause an alteration of the microbiome that damages the intestinal mucosal barrier, leading to increased permeability. B catenin activation of the intestinal endothelial cells prevented barrier disruption [117,118]. Bacteria and their products enter the portal blood, leading to higher concentrations of LPS in the liver, which activates hepatic macrophages through TLR4 signaling and enhances oxidative stress via NADPH oxidase activation [119,120]. Oxidative stress further disrupts the intestinal barrier and a destructive feedback loop is established, promoting liver inflammation and disease progression [121,122].

Obesity and dietary habits alter the human microbiome. *Bacteroides* dominate in high-fat based diets (HFD), while *Prevotella* is prevalent with plant-based polysaccharide diets [123,124]. Studies have shown an increased abundance of bacteria from the Proteobacteria phylum, especially from the Escherichia genus, in individuals with MASLD and MASH compared to healthy individuals [125]. An increased abundance of the *Bacteroides genus* was also described in MASH [113,126]. A study based on biopsy-proven MASLD in Asian patients demonstrated increased levels of primary bile acids and propionate in the stools of non-obese patients with advanced fibrosis. *Veillonellaceae* was the dominant family found in non-obese individuals with MASLD and six genera of this family, *Megasphaera*, *Veillonella*, *Dialister*, *Allisonella*, *Anaeroglobus*, and *Negativicoccus*, were responsible for propionate overproduction, which may be one of the factors leading to the progression to MASLD. A decrease in the *Ruminococcaceae* genus was also associated with fibrosis in MASLD non-obese patients, suggesting that these genera are protective in MASLD progression [127]. An analysis of the gut microbiome of patients with biopsy-proven MASLD found an abundance of *Parabacteroides distasonis* and *Alistipes putredenis* species in MASLD patients. *Prevotella copri* was associated with increased intestinal permeability and MASLD progression [128]. On the contrary, an experimental murine study reported that Bifidobacterium pseudolongum can prevent MASLD-associated HCC [3]. Several studies have examined diet-induced changes in gut microbiota in mouse strains fed a high-fat or high-sucrose diet. However, variability in experimental design and quantitative assessment has made it very difficult to assess the reproducibility of results. The ratio of the *Firmicutes* phylum to the *Bacteroidetes* phylum is consistently increased after high-fat diets according to an extensive meta-analysis [129]. Moreover, microbiota of cecum and feces from animals on HFDs showed increased levels of ethanol production compared to mice fed with normal diets [130] that may be an additional factor for MASLD progression. Interestingly, high-fat diets in mice led to malonaldehyde modifications of Gram-negative bacterial end products, and reduced defensin expression. These changes induced bacterial cytolysins and may affect antimicrobial defense mechanisms in the gut [31]. On the contrary, treatment with the butyrate-producing bacterium, *Kineothrix alysoides*, attenuated liver fat deposition by restoring gut eubiosis [131].

### 2.8. Microbiome-Related Compounds Implicated in the Pathogenesis of MASLD

#### 2.8.1. Short-Chain Fatty Acids (SCFAs)

SCFAs are fatty acids with less than six carbons [132]. They are produced from anaerobic fermentation by gut microbiota, mostly from fibers. They promote the normal function of the intestinal barrier and support gluconeogenesis and lipogenesis [133,134,135]. Most published data favor an inflammatory effect, but results from experimental studies are conflicting [136]. Increased fecal levels of acetate and propionate were associated with MASH and hepatic fibrosis [137]. Butyrate, on the other hand, has anti-inflammatory properties and may protect from diet-induced obesity, liver fat deposition, and insulin resistance [138,139]. Murine models of MASLD have demonstrated that supplementation of butyrate led to decreased liver and adipose tissue inflammation and reduced endotoxin-releasing bacteria in the gut microbiome [133,140]. Human studies are conflicting. A study reported that circulating butyrate in patients with cirrhosis were inversely related to inflammatory markers, but in another study, increased levels of butyrate were associated with mild or moderate MASH [141,142].

#### 2.8.2. Choline

Choline is needed for the synthesis of phosphatidylcholine and the production of acetylcholine and very low-density lipoproteins (VLDLs) [132]. Choline deficiency therefore results in decreased production of VLDLs, leading to the accumulation of triglycerides in the liver [143,144]. However, choline may also induce MASLD. Certain gut microbes convert choline into trimethylamine (TMA), which is further oxidized by hepatic monooxygenases to trimethylamine N-oxide (TMAO), which is damaging to the liver [132,143,144]. High rates of conversion of choline to TMA by the gut microbiota lead to a relative deficiency of choline, resulting in liver inflammation and lipid deposition [145,146]. In addition to this indirect damage, TMAO may directly harm the liver through the reduced activity of CYP7A1 and CYP27A1 enzymes promoting hepatic steatosis, as proven in preclinical and clinical studies [147,148]. A Chinese study with biopsy-proven MASLD showed higher levels of TMAO, correlated with disease severity [149].

#### 2.8.3. Bile Acids

Bile acids are synthesized in the liver from cholesterol. They are endogenous ligands of nuclear receptors that regulate lipid metabolism [150,151]. The bile acid receptor, G protein-coupled bile acid receptor 1 (TGR5), integrates glucose, lipid, and energy metabolism [152]. TGR5 activates PPAR-a and PPAR-γ coactivator 1 alpha (PGC-1a) to increase mitochondrial oxidative phosphorylation, inhibiting NF-kB-mediated pro-inflammatory cytokine production [153,154].

Bile acids are involved in the pathogenesis of MASLD through modulation of the gut microbiome. They are classified as primary (e.g., cholic acid and chenodeoxycholic acid) and secondary bile acids (e.g., deoxycholic and lithocholic acid) [150]. Upon secretion in the intestine, they are involved in the absorption of lipids and lipid-soluble vitamins and prevent bacterial overgrowth, maintaining the composition of the microbiome. Changes in the secretion of bile acids may result in alterations of the microbiome, leading to steatosis and hepatitis [155]. Increased levels of chenodeoxycholic acid were identified in MASH patients and correlated with histological severity and degree of fibrosis [156]. This is probably mediated through the binding to receptors, such as the nuclear farnesoid X receptor (FXR) and TGR5 [157]. FXR activation under homeostatic conditions reduces hepatocyte cholesterol accumulation, VLDL production accompanied by promotion of free fatty acid oxidation, and improvement of insulin [158]. The interaction of bile acids with FXR induces FGF19 secretion, which is a negative regulator of bile acid synthesis in hepatocytes [159]. It has been hypothesized that gut dysbiosis modifies the primary and secondary bile acid balance, leading to disruption of FXR signaling and the promotion of lipid and glucose dysregulation [157,160].

Altered bile acid metabolism in MASLD triggers ROS production and affects lipid metabolism by increasing lipid peroxidation. Moreover, they mediate mitochondrial dysfunction and impair enzymes of the respiratory chain [161,162,163].

Detailed descriptions of bile acid and MASLD were recently published [158,164,165].

### 2.9. Adipocentric Theory

Insulin resistance [166] and adipose dysfunction [167] are implicated in the development of MASLD in the group of individuals with metabolically unhealthy obesity (MUO). However, as mentioned before, not all obese patients develop MASLD, diabetes, or dyslipidaemia [168], a situation designated as metabolically healthy obesity (MHO). It has been proposed that this group is protected from metabolic consequences by efficient fat storage in healthy expandable adipose tissue [169,170]. On the other hand, MASLD is being increasingly diagnosed in lean individuals (metabolically unhealthy lean, MUL). It is plausible that this group has a limited capacity for adipose expansion and restricted fat storage capability.

Adipose tissues and the liver can adapt to an energy surplus by allowing for triglyceride storage up to a certain level without significant consequences. When lipid storage in white adipose tissue (WAT) exceeds that point, inflammation, aberrant adipokine secretion, and liver fat deposition follows. Initially, hepatocytes accumulate triglycerides, leading to a relatively benign fatty liver. Amelioration of obesity-induced adipose tissue dysfunction promoted by healthy adipose tissue expansion and brown adipose tissue activation could prevent triglyceride accumulation in the liver [171].

Secretion of adipokines plays a central role in this adipocentric theory. Adiponectin is the better-studied adipokine in MASLD. It is produced by adipocytes and negatively correlates with insulin resistance. It is an anti-inflammatory factor by repressing the production of pro-inflammatory cytokines and by inducing the production of IL-10 [172]. It decreases lipid deposition in the liver and the influx of fatty acids to the liver [173]. Importantly, patients with MASLD had reduced expression of adiponectin in their livers [174]. A large meta-analysis of 28 reports on MASLD patients showed that patients with MASH had very low adiponectin serum levels [175]. This was also true in lean MASLD patients, reflecting the interplay between the adipose tissue and the liver [176].

A study of leptin and its receptor demonstrated elevated serum leptin levels in patients with biopsy-proven fatty liver but lower concentrations of its receptor, possibly indicating increased peripheral resistance to leptin action [177]. Increased leptin levels were confirmed in another study in prediabetes patients with or without MASLD. It was suggested that increased leptin was accompanied by peripheral or central leptin resistance and increased steatosis and insulin resistance [178].

Resistin, another adipokine, also has an important role in steatosis, insulin resistance, and inflammation. Resistin activates the NF-kB pathway, leading to overproduction of pro-inflammatory cytokines. In murine models on HFD, resistin led to abnormal mitochondrial function through the AMP-activated kinase/PGC-1 pathway [179].

A nuclear receptor that is important in the adipocentic theory is the peroxisome proliferator-activated receptor gamma (PPARγ). It is crucial in the modulation of lipid and glucose metabolism [180]. PPARγ is mostly expressed in white and brown adipocytes and macrophages, and its activation favors lipid accumulation by enhancing adipogenic and lipogenic gene expression. PPARγ activation also makes adipocytes sensitive to insulin, increasing lipid synthesis [178,180].

Leucine-rich alpha-2-glycoprotein 1 (LRG1) is a glycoprotein that has increased in the serum of obese individuals. Similarly, LRG1 increased in serum and adipose tissues in murine models of obesity. Deletion of Lrg1 led to decreased body weight and improvement in insulin resistance. Increased LRG1 secretion from adipose tissues increased de novo lipogenesis and decreased fatty acid oxidation, contributing to the development of MASLD. Therefore, LRG1 seems to act as a detrimental adipokine that promotes obesity-induced MASLD and aggravates systemic insulin resistance [181].

In addition to adipokines, adipose tissue is a source of exosomes, which are implicated in glucose and lipid metabolism [182,183] and participate in the pathogenesis of MASLD. Exosomes produced from adipose tissues communicate with other tissues via circulation. Murine studies have shown that injection of exosomes from macrophages of the adipose tissue of obese mice into lean mice induced insulin resistance [184]. Plasma exosomes in obese patients with MASH were higher than in healthy obese patients [185] indicating that adipose exosomes may be a decisive factor that separates metabolically unhealthy obesity from metabolically healthy obesity. The signals transmitted by exosomes include extracellular microRNAs (miRNAs) that can regulate gene expression in remote organs such as the liver. It has been demonstrated that knockout mice deficient for the miRNA-processing enzyme Dicer in their adipose tissue have decreased levels of circulating exosomal miRNA [186].

A detailed description of dysfunctional adipose tissue in NAFLD has been published [187].

### 2.10. MirRNAs in MASLD

MASLD is associated with abnormalities of hepatic miRNA expression at every stage of the disease. Specific miRNAs are involved in the progression of MASLD to MASH. MiR-34a is increasing with MASLD progression. Several miR-34a targets were identified in MASLD, reducing fatty acid oxidation and inducing lipogenesis and cholesterol synthesis. Among the target genes are SIRT1 and PPARγ, leading to activation of HSC and fibrosis [188].

The most extensively studied miR in MASLD is miR-122, which increases during early MASLD and declines with progression toward MASH. Evidence on the role of miR122 in lipid metabolism is conflicting. MiR122 may be either pro-steatotic or anti-steatotic, as both negative and positive effects on cholesterol synthesis and fatty acid oxidation have been reported. Loss of miR-122 has been involved in fibrosis progression and protects against hepatic inflammation by targeting RELB, a member of the NF-κB family of transcription factors. An additional target of miR122 is cell death-inducing DFFA-like effector c (CIDEC), a protein connected to lipid droplet formation, which is implicated in the progression of MASLD to MASH [189,190]. In a murine model, pharmacological activation of hepatic miR-122 expression and secretion with an agonist of the RAR-related orphan receptor A (RORA) ameliorated hepatic steatosis [191].

MiR-21 also increased with MASLD progression. Results are conflicting. Studies have demonstrated an implication of miR-21 in early MASH by targeting PPARa, while other studies indicate that miR-21 is implicated in later MASLD stages only. miR-21 reduces hepatic insulin sensitivity and promotes steatosis and fibrosis by activating HSCs [192,193].

A detailed description of miRs in MASLD has been recently published [194].

### 2.11. The Mode of Hepatocyte Death in MASLD

The dominant mechanism of death in MASLD has not been clarified. Increased TUNEL positivity and increased caspase-3/7 expression was found in biopsy material from patients, which argues in favor of apoptosis as the main mechanism of death. A further argument for apoptosis is that administration of the pan-caspase inhibitor, Emricasan, ameliorated liver damage and fibrosis in a murine model [195]. However, early clinical trials in MASH patients [195,196] and a large randomized clinical trial of Emricasan did not show significant clinical improvement [197]. However, several investigators still support the idea that apoptosis is the predominant mode of cell death, with involvement of both intrinsic, through lipotoxicity, and extrinsic pathways.

Nonetheless, other forms of cell death seem to be implicated, including necroptosis and pyroptosis of hepatocytes. Pyroptosis, which is a form of programmed cell death driven by inflammasome activation, has been mainly demonstrated in animal models of MASH [198,199] and less reliably in patients with MASH [200]. It manifests as cellular distension until rupture of the cell membrane and spillover of cellular contents, which activate an intense inflammatory response. Classical pyroptosis is also regulated by gasdermin D (GSDMD) [201]. Expression of Gasdermin D and its fragment GSDMD-N protein was significantly upregulated in liver biopsies of MASH patients and correlated with disease activity and fibrosis. On the contrary, GSDMD-/-mice did not develop steatohepatitis and fibrosis, suggesting a critical role of GSDMD-associated pyroptosis in promoting MASH [200].

Evidence of necrotic cell death, in addition to apoptosis of hepatocytes, has been reported in MASH. The common mechanism between necrosis and apoptosis in MASH is the accumulation of free cholesterol in MASH patients [202,203] through activation of c-Jun N-terminal kinase 1 (JNK1) [204].

DAMPs, such as high-mobility group box 1, adenosine triphosphate, and mitochondrial DNA, liberated during necroptosis and pyroptosis, are recognized by pattern recognition receptors (PRRs), like TLRs, and nuclear receptors (NLRs), such as nucleotide-binding domain, leucine rich-containing family, pyrin domain-containing-3 (NLRP3) inflammasome on KCs, and dendritic cells initiating inflammation and cytokine release [205,206]. PAMPs derived from gut microbiota, such as LPS, are recognized by TLR4, also triggering inflammation.

A recently described form of programmed cell death seems to be an important mechanism of hepatocyte death in MASH. Ferroptosis is an iron-dependent type of non-apoptotic cell death, which is driven by the accumulation of lipid peroxides and ROS [207,208,209]. Targeting ferroptosis in a murine model of MASH improved the accumulation of free lipid droplets and repressed lipotoxicity [210]. Importantly, all the characteristics of ferroptosis have been demonstrated in the majority of MASH patients [211]. It is now evident that there is a strong relationship between ferroptosis and MASLD [212,213,214]. Ferroptosis is a stronger inducer of inflammation compared to necroptosis in MASH [215]. Interestingly, a reduction in intrahepatic polyunsaturated fatty acids (PUFA) caused by deficiency of fatty acid transport protein 5 (FATP5) led to the upregulation of SREBP1/stearoyl-CoA desaturase 1 (SCID1) to increase monounsaturated fatty acids (MUFA) and repression of ferroptosis. Ultimately, an amelioration of MASH was observed [216]. The ferroptosis inhibitor liproxstatin-1 alleviated hepatocyte apoptosis, pyroptosis, and necroptosis in a mouse model of MAFLD [217], suggesting that ferroptosis may be the initial trigger underlying the other forms of hepatocyte death. Thymosin beta 4 improves the liver fibrosis and reduces inflammation in MESH by inhibiting the GPX4-mediated ferroptosis [218]. TRIM59, a member of the TRIM family, enhances ferroptosis by increasing GPX4 ubiquitination. The overexpression of GPX4 was reported to reverse the pathogenic effects of TRIM59 in MASLD [219,220]. The specific effects of these forms of death on the function of liver macrophages will be discussed in Section 3.

The mechanisms of ferroptosis in MASLD and its contribution to the progression of MASLD have been extensively reviewed in recent publications [77,221].

**Figure 1:**
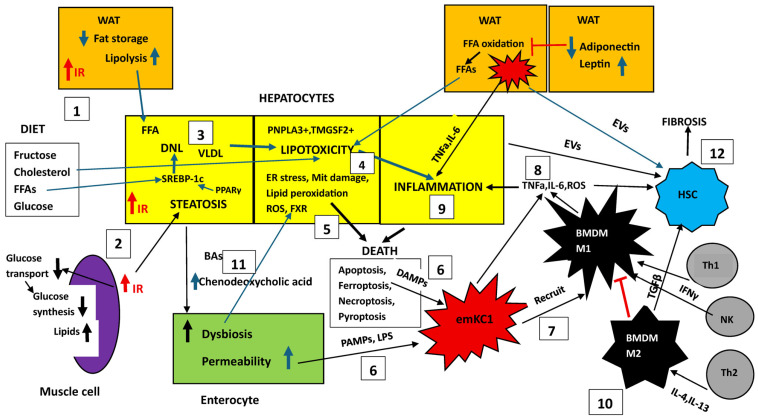
A diagrammatic presentation of MASLD pathogenesis. Diet factors such as fructose and glucose, obesity, and genetic abnormalities initiate MASLD (1). The characteristic increase in insulin resistance (2) in these patients is due to increased lipid production in either the hepatocytes or the white adipose tissue and the skeletal muscles. Increased FFAs in the hepatocytes are supplied either through diet and the intestine or from the white adipose tissue through increased lipolysis (3). Increased de novo lipogenesis leads to fat deposition in hepatocytes, which induces lipotoxicity (4). ER stress, increased ROS production, mitochondrial damage, and lipid peroxidation are the results of lipotoxicity (5), leading to hepatocyte death and release of DAMPs that activate Kupffer cells (6), leading to an activation of an immune cascade including recruitment of bone marrow-derived macrophages (7) and production of pro-inflammatory cytokines (8). Aggravation of inflammation (9) and trans-differentiation of HSCs leading to fibrosis is the end result of the immune activation (12). Anti-inflammatory M2-polarized macrophages may inhibit this cascade (10), but M2 macrophages are pro-fibrotic as well through TGFβ production. The intestine participates by a disturbed microbiota that increases intestinal permeability and liberation in the portal blood of RAMPs that further activate macrophages (6). Bile acids, particularly chenodeoxycholic acid (11), are implicated in dysbiosis and the activation of the nuclear receptor FXR in the hepatocytes promoting lipotoxicity. BA: bile acids; BMDM: bone marrow-derived macrophage; DAMPs: damage-associated molecular patterns; DNL: de novo lipogenesis; EVs: extracellular vehicles; FXR: farnesoid X receptor; FFAs: free fatty acids; HSCs: hepatic stellate cells; IR: insulin resistance; LPS: lipopolysaccharide; PAMPs: pathogen-associated molecular patterns; PPARa: peroxisome proliferator–activated receptor alpha; ROS: reactive oxygen species; SREBP1c: sterol regulatory element binding protein 1c; WAT: white adipose tissue.

A diagrammatic presentation of MASLD/MASH pathogenesis is presented in Figure 1.

### 2.12. Treatment of MASLD/MASH

Detailed studies of MASLD pathophysiology have identified molecular targets that have the potential to attenuate the progression of the disease. Improvement of the disturbed function of the adipose tissue may reduce steatosis and ameliorate inflammation and fibrosis. FGF21 analog is an insulin sensitizer acting on adipose tissue through the receptor complex of b-klotho and one of the FGF receptors [222], protecting against MASLD [223]. It should be noted that FGF21 is induced by cold exposure in brown and white adipose tissue (WAT), increasing browning in inguinal white adipose tissue [224,225].

Additional drugs suggested for MASLD include drugs targeting bile acid regulation, such as FXR agonists and antagonists, and drugs that include PPAR agonists, glucagon-like pepetide-1 (GLP-1) agonists, and thyroid hormone receptor beta (THR-β) agonists. In addition to anorexigenic actions, GLP-1 treatment downregulated the expression of inflammatory cytokine genes, such as IL-6, TNFa, and CCL2, in adipose tissue by suppressing the NF-kB pathway [226]. Other studies showed that GLP-1 analogs improve insulin sensitivity in adipose tissue [227,228]. A clinical study showed that the GLP-1 analog liraglutide decreased body weight, hepatic triglyceride content, and visceral fat in obese people [229]. Semaglutide also induced MASH resolution in humans [230]. The synergistic effects of GLP-1 on adipose tissue and the liver reduce adipose tissue inflammation and liver steatosis.

PPARγ agonists such as pioglitazone are used for treatment of diabetes mellitus [180]. Pioglitazone increases insulin-induced repression of lipolysis in diabetes [231]. It also improves adipose tissue insulin resistance in patients with MASH, reducing hepatic triglycerides, inflammation, and visceral fat [232]. Moreover, pioglitazone increases adiponectin levels and improves liver histology in patients with MASH [233].

Modulation of gut microbiomes may also be used to treat MASLD. Probiotics seem to improve lipid profiles in patients with MASLD [234], but most studies were performed on animal models [235]. The most widely used probiotics include *Bifidobacterium* and *Lactobacillus* species or a combination of them, but new probiotics, such as *F. prausnitzii* and *A. muciniphila*, may show better results. Treatments targeting microbiota that are based on the administration of different probiotics have recently been reviewed [236].

Resmetirom is a selective THR-β partial agonist that mostly acts on the liver. It has reduced affinity for THR-α, which is expressed in the heart and bones. Therefore, the risk of cardiovascular and skeletal side effects is minimal. Resmetirom activates THR-β and heterodimerizes with the retinoid X receptor after transporting it to hepatocytes using the organic anion transporting polypeptide 1B1. The heterodimer transcripts genes implicated in lipid metabolism, imitating the beneficial effects of endogenous T3 [237,238].

The specific effects of these drugs on KCs and BMDMs will be discussed in Section 3. Recent reviews on drug treatment of MASLD/MASH have been published [239,240,241,242,243].

## 3. Kupffer Cells in MASLD/MASH

KCs are resident liver macrophages located on the luminar side of the sinusoids. They comprise approximately 30% of liver sinusoidal cells [244]. They are the first line of defense against microbes [245,246] and phagocytose debris arriving at the sinusoids through the portal or hepatic artery blood [247,248]. They also participate in iron recycling through phagocytosis of aged red blood cells [249,250,251]. Moreover, they are involved in lipid metabolism [252] and are responsible for maintaining immunological tolerance [253,254,255]. Scavenger receptors are expressed on the KC surface under normal circumstances, which allows them to recognize and clear apoptotic cells and immune complexes. KCs also express an extensive range of pattern recognition receptors (PRRs), including toll-like receptors (TLRs), nucleotide oligomerization (NOD)-like receptors, and retinoic acid-inducible gene I (RIG-I)-like receptors. These receptors allow KCs to recognize and eliminate invading foreign pathogens [256].

Kupffer cell development depends on their interplay with HSCs, which sustain KC identity through a cross-talk between ALK1 (activin receptor-like kinase), expressed in KCs, and BMP 9/10 (bone morphogenic proteins), expressed in HSCs [257,258]. HSCs also produce IL-34 and colony-stimulating factor-1 (CSF1), which are necessary for KC survival and proliferation [259].

KCs detect danger signals such as cholesterol crystals and free fatty acids in MASLD [260], but also PAMPs coming through a defective intestinal barrier. KCs respond to these signals by secreting pro-inflammatory cytokines and chemokines [261,262] that recruit peripheral monocytes and neutrophils via lipocalin 2 [263].

### 3.1. Liver Macrophage Heterogeneity in the Healthy Liver

In early studies, all macrophages expressing general markers were classified as KCs. The majority of macrophages in the healthy liver are KCs originating during embryogenesis from yolk-sac macrophages and fetal liver monocytes [264].

In the healthy murine liver, KCs are defined as F4/80hiCD11bint cells that express T-cell immunoglobulin mucin 4 (TIM4), C-Type Lectin Domain Family 4 Member F (CLEC4F), and Vset and immunoglobulin domain containing 4 (VSIG4) [17,265], whereas monocyte-derived macrophages tend to have a CD11bhi F4/80int phenotype and express CX3C motif chemokine receptor 1 (CX3CR1) and C-C chemokine receptor type 2 (CCR2) [265]. In mice, there is very little supplementation by bone marrow-derived macrophages to the pool of resident KCs [266].

Recruited bone marrow-derived monocytes (BMDM) give rise to self-renewing and fully differentiated KCs that secrete cytokines and chemokines, such as TNF-α and chemokine C-C motif ligand 5 (CCL5), which participate in the subsequent recruitment of immune cells [267]. Therefore, two subsets of KCs are identified in the healthy murine liver, namely BM-derived KCs (moKCs) and embryo-derived KCs (Em-KCs) [17,68,267,268]. MoKCs comprise 20–40% of total KCs [269]. Em-KCs can remove apoptotic cells, aged red blood cells, and pathogens and account for the majority of the healthy KC pool, whereas moKCs infiltrate the liver tissue in disease and elicit a pro-inflammatory effect [254,270,271].

In the human liver, embryonic Kupffer cells (emKCs) express CD49a, which is not expressed in bone marrow-derived Kupffer cells (moKCs). CD49a can be used to distinguish those two subpopulations of KCs. Human emKCs express high levels of pro-inflammatory TNFa and IL-12 cytokines but also the anti-inflammatory IL-10 cytokine, indicating that they have a dual role in inflammation, in contrast to moKCs, which express low levels of these cytokines. An additional difference between these two subpopulations is the fact that LPS does not increase the expression of these cytokines in emKCs, but instead, their expression is highly upregulated by LPS in moKCs. EmKCs seem to be functional in homeostasis, and moKCs become functional in cases of injury, similarly to the murine liver [272].

Two smaller subsets of macrophages exist in the murine liver, namely the liver capsule macrophages (LCMs) and the central vein (CV) macrophages located near the CV. Together they comprise 10% of the total hepatic macrophage. In mice, LCMs express pan-macrophage markers such as F4/80 and CD64, but do not express KCs markers such as VSIG4, TIM4, and CLEC4F. Instead, they express CX3CR1 and CD207. In humans, markers of capsule and CV macrophages have not been determined [257,273,274].

Em-KCs are more heterogeneous in the murine liver. A previous study identified a radiation-resistant Em-KC subgroup in a mouse model [275]. Two distinct populations of Em-KCs, namely, KC-1 and KC-2, have been identified which differ in the expression of many genes and proteins. KC-2 can improve lipotoxicity-induced oxidative stress of hepatocytes through the expression of CD36 and can also reverse hepatocyte-mediated CD8+ T-cell dysfunction [276,277]. KC1s are low in CD206 and Endothelial Cell-Selective Adhesion Molecule (ESAM)-negative while KC2s are high in CD206 and ESAM+. KC1s express the KC markers’ colony-stimulating factor-1 receptor (Csf1r), TIM4, CLEF4F, and F4/80. In contrast, KC2 expresses the markers CD36, lymphatic vessel endothelial hyaluronan receptor-1 (LYVE1), ESAM, and CD206. KC-1 also expresses CD170, which is involved in immune regulation, while KC-2 expresses lipid metabolism-associated genes [276,278,279,280,281].

In healthy human livers, hepatic macrophages can be classified as CD68+MARCO+Timd4+ and CD68+MARCO-Timd4− subsets [282,283]. The CD68+MARCO+Timd4+ cells were considered as resident KCs expressing genes involved in immune tolerance, whereas the CD68+MARCO-Timd4− cells resembled the pro-inflammatory mouse moKCs, with a higher expression of pro-inflammatory genes [284]. Resident KCs and recruited macrophages may be polarized into M1 and M2 according to their function. LPS and interferon-γ induce M1 macrophages, whereas IL-4 and IL-13 lead to M2 polarization. M1 macrophages produce pro-inflammatory cytokines, such as IL-1b and TNF-a, whereas M2 macrophages produce anti-inflammatory factors, such as IL-10 and transforming growth factor-b (TGF-b). A number of intermediate phenotypes have been reported between the two polarized phenotypes. Thus, M2 macrophages can be further classified into M2a, M2b, M2c, and M2d subtypes based mostly on their polarization stimuli [285]. M2a are macrophages alternatively activated by IL-13 or IL-4. The M2b type is stimulated by IL-10 or IL-1RA. M2c macrophages are stimulated by IL-10 or glucocorticoids, and M2d macrophages are stimulated by adenosines or IL-6 [286]. Detailed descriptions of M1 and M2 macrophage markers and differences between mouse and human M1 and M2 macrophages have been published [287].

### 3.2. Liver Macrophage Heterogeneity in MASLD

In an HFD model of MASLD, KC-1 cells differentiated into pro-inflammatory phenotypes were involved in an interplay with invariant natural killer T (iNKT) cells. IL-10 expression was unaffected by a high-fat diet but impaired by iNKT cell ablation. CD206 knockdown also reduced IL-10 expression [288].

EmKCs are diminished in different murine models of MASLD, and the severity of this reduction seems to be inversely related to disease stage [265]. EmKCs are replaced by bone marrow-derived macrophage (BMDMs) subsets. They include the already mentioned MoKC, CCR2-dependent lipid-associated macrophages (LAM) which express osteopontin [278,280,289], pro-inflammatory high-CCR2 high-Ly6C recruited macrophages [278,279,290,291] and low-CX3CR1+Ly6C macrophages [292]. In humans, high-CD14 CD16-, high-CD14 low-CD16 and non-classical low-CD14 high-CD16 BMDMs have been described [293]. The initiating stimuli of the differentiation programs of BMDMs in MASLD, and the functional role of BMDM subpopulations, with the exception of LAMs, require further research [289,294].

KC renewal is impaired in MASH, further promoting the increased recruitment of BMDMs [295]. There are other possible explanations for the reduction in KCs in MASLD. Collagen deposition and the remodeling of the liver architecture after liver injury may lead to the loss of cellular contacts necessary for the survival of KCs, leading to their death [259,296]. In support of this hypothesis, the loss of fenestrations of LSECs that happens in MASLD impairs their contact with KCs [297]. Moreover, the production by HSCs of BMP9, which is vital for the survival of KCs, is greatly reduced due to transdifferentiation of HSCs into myofibroblasts during MALSD progression [297,298]. This communication is bidirectional. It has recently been demonstrated that KCs inhibit HSC activation through the secretion of exosomes containing miR-690, but miR-690 is significantly reduced during MASLD [299]. This idea is also supported by a model suggesting that the loss of emKCs is in fact a so-called “altruistic death”, an event that facilitates the recruitment of BMDMs, which are better equipped to handle the damage [300]. The mechanisms of moKC recruitment have also been clarified. EmKC death liberates TNFa and IL-1b, which activate HSCs and LSECs, leading to the expression of genes implicated in monocyte recruitment such as Ccl2, Ccl7, and Cxcl10, and adhesion molecules that facilitate the catchment and diapedesis of monocytes such as Vcam1, Selectin, and Icam1. Once recruited to the liver, KC identity is printed to them by signals produced by LSECs and HSCs [301]. Despite the identification of the origin and profile of emKCs and moKCs in MASLD, the specific function of these cells in the progression of the disease is unclear. In a disease with so many degrees of inflammation and fibrosis, emKCs and moKCs may function differently according to the stage of the disease [302,303].

In at least some models of MASH, moKCs acquire, with time, TIM4 expression and become indistinguishable from emKCs. The opposite has also been suggested in a model of fibrosis, where emKCs may lose TIM4 expression and be converted into moKC-like cells, but this is not confirmed in other models. The presence of a hybrid LAM/KC subpopulation has been described in the diseased liver, but the ontogeny of these cells is not clear, and their presence in MASLD has not been verified [274,304].

**Figure 2:**
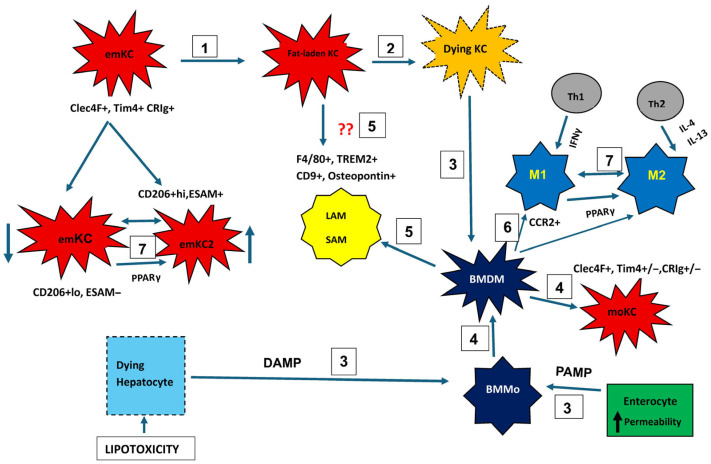
Macrophage role in MASLD. Embryonic resident KCs store lipids at the initial stages of MASLD and gradually lose their ability to self-renewal (1). During disease progression, KCs die by apoptosis, necroptosis, pyroptosis, or ferroptosis (2) and are mostly replaced by monocyte-derived KCs, which are recruited by DAMPS released by the dying KCs and hepatocytes (3). Recruited monocytes are transformed either into bone marrow-derived macrophages or monocyte-derived KCs that cannot be distinguished from the resident KCs (4). Simultaneously, a distinct population of LAMs emerges in connection to steatosis or as SAMs in the fibrotic areas. They are derived from bone marrow-derived macrophages (5). However, fat-laden KCs may participate in the production of LAMs (5), although this has not been proven. CCR2-expressing M1 polarized pro-inflammatory macrophages infiltrate the liver and promote inflammation (6) but also switch the balance toward anti-inflammatory M2-polarized cells through activation of the PPARγ nuclear receptor (7). Among resident KCs, steatosis and the activation of the PPARγ favors the production of anti-inflammatory KC2s whose exact role in the resolution of the disease has not been elucidated. However, the question remains: What is the factor that switches relative benign hepatic steatosis into progressive lipotoxicity? emKC: embryonic Kupffer cells; BMMo: bone marrow-derived monocytes; BMDM: bone marrow-derived macrophages; moKC: monocyte-derived Kupffer cells; LAM: lipid-associated macrophages; SAM: scar-associated macrophages; DAMPs: damage-associated molecular patterns; PAMPs: pathogen-associated molecular patterns. ??: not confirmed in humans.

A diagrammatic presentation of liver macrophages in MASLD can be seen in Figure 2.

### 3.3. KCs in MASLD

Several reports have established that Kupffer cells have a fundamental role in the development of MASH through TLR-4 signaling [68,305]. KCs also promote hepatocyte fat deposition via the IL-1β-dependent suppression of PPAR-α activity [306,307,308]. In obesity, increased PAMPs originating in the intestine, such as LPS and bacterial DNAs, directly activate TLRs in KCs [199,261]. Specifically in MASH, TLR4 expression in KCs and moKCs is higher compared to other TLRs [309]. LPS binding to TLR4 triggers MAPK, p38, and NF-kB signaling [310,311,312] to induce pro-inflammatory cytokines such as TNFα, IL-1b, and IL-12 and chemokines such as CCL2 and CCL5 secretion that promote local inflammation.

### 3.4. Activation of Kupffer Cells

Mice lacking moKCs show less steatosis, hepatic inflammatory cell infiltration, and fibrosis [313]. Moreover, destruction of KCs in the early stages of MASLD will decrease the extent of liver damage [74]. Several mechanisms of KC activation have been elucidated.

Free fatty acid (FFA) activation in KCs leads to NLRP3 inflammasome activation. Experimental stimulation of KCs with palmitic acid (PA) initiates the formation of the mtDNA-NLRP3 inflammasome complex, which is inhibited by deletion of NLRP3 [314]. In addition, increased FFAs lead to overproduction of TNFα through TLR4 on KCs acting as a sensor for FFAs [260]. Not all fatty acids are equally effective in KC activation. Saturated fatty acids strongly induce the production of TNF-α and IL-6. On the contrary, unsaturated fatty acids, such as docosahexaenoic acid, induce the expression of M2 polarization markers, including IL-10 [315]. In MASLD, the KC scavenger receptor 1 and the fatty acid transporter CD36, which mediate the uptake of modified low-density lipoprotein, are considered important in pathogenesis [316,317]. Activated, fat-laden KCs have dysregulated lipid metabolism and recruit other immune cells due to their inflammatory phenotype [318].

Hepatic free cholesterol overload results in lipotoxicity [62]. Cholesterol and fat synergistically may modify the phenotype of macrophages [319]. Furthermore, an immunohistochemical study of the liver macrophage marker Iba-1 has shown that the number and staining area of macrophages positively correlated to increasing dietary cholesterol [320]. Macrophages from mice and humans do not synthesize cholesterol de novo. Therefore, KCs can only take up cholesterol from lipid droplets of dead fatty-laden hepatocytes or by binding of oxidized low-density lipoproteins (ox-LDL) to CD36 or scavenger receptors [62,321,322]. Dysregulated uptake of ox-LDL may lead to deposition of cholesterol in the lysosomes and initiation of inflammatory responses in KCs [323]. Activation of KCs by cholesterol is mediated by the nuclear receptors liver x receptor α (LXRα) and LXRβ [324,325]. Selective stimulation of intestinal LXR results not only in the reversal of cholesterol transport, but also in the promotion of the anti-inflammatory effect of HDL cholesterol through its interaction with the SRB1 receptor, and the switch of KCs from M1 to the M2 polarization, reducing the oxidative stress in hepatocytes [326]. Additionally, free cholesterol initiates the expression of sphingomyelin synthesis (SMS1), which is a mediator of diet-induced hepatocyte pyroptosis [327,328]. DAMPs liberated by pyroptotic hepatocytes increase IL-1β and activate NLRP3 inflammasomes in KCs, leading to inflammation in MASH [329]. Furthermore, in a murine model, dietary cholesterol repressed 7-dehydrocholesterol reductase expression in liver macrophages, leading to a pro-inflammatory phenotype followed by steatohepatitis, which was reversible by simvastatin treatment [330]. However, it should be noted that, although cholesterol-induced lipotoxicity leads to oxidative stress, hepatocellular senescence, and lipoapoptosis [331], there is a non-linear relationship between simple steatosis and lipotoxicity. No conclusive evidence has proven that patients with severe steatosis progress to MASH more rapidly than those with a lesser degree of steatosis burden [332].

Apart from the classical activators of KCs described above, a number of additional factors also activate KCs [305].

Macrophage scavenger receptor 1 (MSR1, CD204) is responsible for lipid uptake in KCs and is associated with the severity of MASLD and MASH in patients. Its depletion abrogates lipid accumulation in KCs and their pro-inflammatory polarization [316].

STING is a pathogen recognition receptor (PRR) localized on the surface of KCs [333,334,335] which is implicated in the progression of inflammation and connective tissue proliferation in the liver by activating KCs and HSCs [336,337]. Patients with mild or advanced fibrosis in MASH have a higher number of STING+/p-TBK1+ cells in the liver than healthy controls [338], indicating that the STING/TBK1 signaling pathway is activated in KCs during progression of MASH.

Moreover, liver sinusoidal endothelial cells (LSECs) lose their fenestrations and undergo capillarization in the course of MASH progression, releasing inflammatory molecules that activate adjacent KCs and exacerbate inflammation [339]. Copper and lipids independently participate in the pathogenesis of dyslipidemic diseases [340]. Increased serum copper is inversely related to MASLD and may be protective against MASH development [341,342]. Copper–fructose interaction-induced hepatic steatosis is eliminated by KC depletion [343,344], indicating that KCs are involved in the copper modulation of MASLD [345].

Apoptotic hepatocytes accumulated in human and experimental MASH are associated with impaired efferocytosis and loss of TIM4 in KCs, leading to increased profibrotic activation of HSCs and acceleration of the progression to fibrotic MASH. Genetic restoration of macrophage Timd4 promoted the clearance of apoptotic hepatocyte and decreased HSC activation and fibrosis.

### 3.5. Lipid-Associated Macrophages (LAMs) in MASLD/MASH

LAMs were first described in adipose tissue, where they are involved in the degradation of lipids via lipoprotein lipase and lipid metabolism via the fatty acid transporter CD36 and the fatty acid binding proteins 4 and 5 (Fabp4, Fabp5) [289,346]. In the healthy liver, their numbers are small, but upon the onset of inflammation, LAMs comprise up to 50% of total liver macrophages. There is some confusion around their definition. They have also been referred to as scar-associated macrophages or SAMs [74,284,347,348], as Trem2+ macrophages [349,350,351], or as osteopontin (ssp1) + macrophages [352]. LAMs express a high level of the chemokine osteopontin [280], which is upregulated in human and murine MASH [353,354,355]. Liver LAMs express the common macrophage markers, but do not have KC markers. They are similar to LAMs from adipose tissue [356]. They also express high levels of triggering receptors expressed on myeloid cells 2 (Trem2) and glycoprotein non-metastatic melanoma protein B (Gpnmb), along with Cd9, Spp1, and Clec4d markers [279,280,356]. Trem2 is expressed not only in LAMs and moKCs, but also in KCs after injury, possibly by acquisition of a LAM-like phenotype [278,279,281]. The number of LAMs is directly related to the severity of the disease [280,302]. LAMS are also identical to the recently described MASH-associated macrophages revealed by secretome gene analysis in mouse and human disease. MASH-associated macrophages are distinguished by their high expression of Trem2 and are also associated with disease severity, as are LAMs [281].

BMDMs recruited in the murine MASLD liver can differentiate either into moKCs or into LAMs (Figure 2). The final decision for this transition is possibly dictated by the local signals they come across at the site of recruitment [257]. Injuries in the liver lobule have regions with more intense damage and regions with little or no damage. In MASLD, LAMs are mostly located in areas of steatosis and fibrosis, whereas moKCs are mostly located in areas with less damage [257,280,302]. Other factors that dictate the transition into LAMs include the efferocytosis of dying cells [350,357] and lipid stimulation, which in vitro has been shown to drive the transition of BMDM toward the LAM phenotype [257]. Moreover, the presence of so-called “find me” signals such as sphingosine-1-phosphate, which are produced by injured hepatocytes, drive the transition into LAM phenotype through the interaction with sphingosine-1-phosphate receptor 1 [350]. After resolution of MASLD, two studies reported that LAMs were dramatically reduced while moKCs were significantly reduced and emKCs increased [295,303].

LAMs are localized around fat-laden hepatocytes [257,280,302]. A common histological feature of MASH is the appearance of hepatic crown-like structures (hCLS), which are in fact a macrophage formation around large lipid droplets or damaged hepatocytes. LAMs have been implicated in the formation of these hCLS [302,358]. Loss of LAMs protected from the formation of hCLS and was associated with increased fibrosis in a model of MASH [302]. Other reports verified that the Trem2 molecule itself plays a protective role in liver damage [359] and fat deposition. Trem2 deficiency increased fat deposition, dyslipidemia, and glucose intolerance in diet-induced obesity [356]. Deletion of Trem2 aggravates MASLD, and Trem2-deficient BMDMs were demonstrated to have pro-fibrogenic potential in vitro [349,351]. It has been suggested that Trem2 mediates macrophage efferocytosis of lipid-laden hepatocytes in MASLD, repressing inflammation in the steatotic liver. Comparison of LAMs between healthy and obese mice showed decreased production of IL1b, TNF, and IL10 in obesity [257]. Therefore, LAMs may be either harmful [257,284,348] or protective [302,352] during MASH progression, and the reasons for this dichotomy remain unknown. On the other hand, genetic deletion of Trem2 synergized with an NK cell-activating agent to inhibit lung cancer growth, suggesting that TREM2+ macrophages repress NK cell accumulation and cytolytic activity. Whether this is also true for MASLD-associated HCC remains to be investigated [357].

Gpnmb gene expression is of particular interest in MASLD, but results are conflicting. It is highly expressed in two BMDM subsets, LAMs and MoKCs [257,360,361]. Overexpression of hepatic Activin A reduced liver steatosis and inflammation and improved insulin sensitivity in parallel with a significant decrease in Gpnmb [362], suggesting that Gpnmb is harmful in MASLD. On the contrary, the overexpression of Gpnmb alleviated fat accumulation and fibrosis in a murine model, but paradoxically, patients with MASH had higher serum soluble GPNMB concentrations compared to patients with simple steatosis [363]. This finding confirmed a previous study where overexpression of Gpnmb in the liver ameliorated hepatic fibrosis in a rat model of MASLD [364]. Moreover, a Gpnmb knockout mouse line developed adipose tissue inflammation, insulin resistance, and liver fibrosis [365]. However, a very recent study seems to corroborate the harmful effect of Gpnmb expression in MASLD. Notably, myeloid-specific Gpnmb deletion led to the retention of emKCs and redirected the recruited BMDMs toward MoKCs, thus blocking the formation of LAMs. This transition significantly reduced steatosis and mildly decreased liver fibrosis in this model [366].

MASLD is not the only liver disease where LAMs are important pathogenetically. LAMs are also recruited in other liver injuries that are not related to fat deposition or abnormal metabolism such as acetaminophen overdose. Interestingly, in this condition, LAMs are derived from both monocytes and KCs. LAM-like KCs express the KC markers Clec4f, Vsig4/CRIg, and Marco, but also the LAM markers Trem2 and CD9, among others [367].

In summary, LAM functions in MASLD are not fully clarified. A generalized deletion of Trem2 or deletion of Trem2 from myeloid cells indicates that these cells are protective in MASLD. In addition, manipulating expression levels of osteopontin in myeloid cells in MASLD corroborates a protective role for LAMs. The results of overexpression of the LAM gene Gpnmb are conflicting, with studies indicating both detrimental and protective effects.

### 3.6. M1/M2 Functional Modifications of KCs and Liver Macrophages in MASLD

As liver steatosis progresses, there is a gradual increase in the M1 polarized macrophages and a reduction in M2 cells, leading to secretion of pro-inflammatory molecules [368]. In the initial stages of MASLD, experimental evidence demonstrated dominant M2 KC polarization, M1 KC apoptosis, and resistance to hepatocyte steatosis and apoptosis [369]. Multiple factors, both intrahepatic and extrahepatic, are responsible for the transition of KCs and BMDMs from M2 to M1 polarization during MASLD development. LSECs and activated HSCs produce pro-inflammatory molecules that switch the polarization of KCs. Obesity and impaired intestinal barrier send increased levels of LPS to interact with TLR4 receptors in KCs, promoting inflammation, which turns the balance of M1/M2 toward M1 polarization [370]. Murine models and in vitro cellular experiments have shown that in the late fibrotic stage of MASH, the protein S100A8 is a DAMP that activates the TLR4 receptor, leading to the production of ROS, activation of NLRP3 inflammasomes, and pyroptosis of KCs. S100A8-stimulated pyroptotic death of KCs resulted in the activation of a human hepatic stellate cell line and increased collagen deposition [371]. Another factor that is responsible for M1 polarization of KCs is the activation of TLR-9 by mitochondrial DNA and intact mitochondria that circulate in the plasma of MASLD patients [372]. Elevated oxidized mitochondrial DNA was also detected in liver biopsies from patients with NASH [373]. Deletion of TLR-9 from KCs led to resistance to M1 activation and switched them to the M2 phenotype [374]. These findings indicate that TLR-9 is a major factor in determining the M1 phenotype in KCs [375]. As a compensatory mechanism, IL10 secreted by M2 KCs promoted selective M1 death, and anti-IL10 antibody administration mitigated the pro-apoptotic effects of M2. The promotion of the M2 phenotype under these conditions destroyed M1 KCs, thus ameliorating MASLD [369].

Diabetes upregulated genes connected to inflammatory cytokine production and increased the M1/M2 macrophage ratio in the liver. Single-cell RNA sequencing analysis of sinusoidal cells showed that diabetes reduced emKCs and increased BMDM-recruited inflammatory macrophages [376].

Hypoxia-inducible factors (HIFs) are implicated in the polarization process of macrophages. HIF-1α favors M1 polarization, whereas HIF-2α promotes M2 polarization. HIF-2a attenuates insulin resistance and represses NLRP3 activation in obesity [377,378,379,380]. Palmitic acid deranged autophagy via HIF-1α activation in macrophages. HIF-1α activation and decreased autophagy stimulated inflammation in macrophages through upregulation of NF-κB activation [381]. In a recent study, HIF-2α mediated a MASH-associated decrease in KC efferocytosis by enhancing lysosomal stress and lysosomal cell death. In BMDMs, in contrast to KCs, HIF-2α promoted mitochondrial ROS production and pro-inflammatory activation. These results suggest that specific macrophage effects of HIF-2α contribute to the pro-inflammatory activation of liver macrophages, leading to the development of MASH [382].

Another factor that favors M1 polarization in KCs and plays a role in MASLD is retinol-binding protein 4 (RBP4). Exosomal RBP4 derived mostly from hepatocytes promoted the M1 polarization of KCs by increasing the activation of NF-κB and ROS accumulation. M1 production of pro-inflammatory cytokines increased FFA uptake and lipogenesis-related genes, such as SREBP-1c, but decreased fatty acid degradation-related genes, such as PPARα, in cellular experiments [383]. KCs are known to produce ROS through the activation of M1 polarization and TLR signaling [384]. KCs are a major source of ROS because hepatocytes have a higher antioxidant capacity than KCs [385]. Several studies have shown the efficacy of antioxidant treatments that prevent progression of fibrosis by reducing ROS [386,387]. Thus, gondoic acid was shown to significantly reduce LPS-stimulated ROS levels and enhance the expression of antioxidant genes in KCs [388]. Similarly, the deletion of myeloid forkhead box O1 (FoxO1) switched the macrophage polarization from the M1 to M2 phenotype and decreased liver macrophage infiltration and inflammation in a murine model of MASH [389].

The three isoforms of PPARs, PPARα, β/δ, and γ are implicated in the different polarization of macrophages in hepatic inflammation [390,391]. Current evidence indicates that activation of PPARγ and PPARβ/δ are necessary for M2 polarization of macrophages [315,392]. Macrophages experience metabolic rewiring in specific inflammatory microenvironments, such as MASLD/MASH, to adapt for specific events such as phagocytosis and cytokine production [239,304,393]. Macrophages demand a lot of energy to execute their functions in inflammation, hence the need for metabolic rewiring. The various phenotypes of macrophages may have different energy needs and different metabolic profiles [394]. Reprogramming usually relates to lipid metabolism, as macrophages are cells capable of extensive lipid processing [395]. In addition to hepatocytes, KCs and BMDMs are rich in lipid droplets, where they are used for the production of bioactive molecules such as prostaglandins and leukotrienes [77,79] that further promote inflammation and immune cell liver infiltration. Immunometabolism studies have shown that the maintenance of several functions of KCs depends on certain metabolites and nutrients [396]. A recent study demonstrated that deletion of CD206hi ESAM+ KCs or inhibition of their fatty acid transporter protein CD36 reversed obesity and steatosis in mice [276]. CD36 and CD206 are both commonly considered to be M2 markers [397]. It has been suggested that glycolysis characterizes M1 macrophages and fatty acid oxidation characterizes M2 cells [398].

### 3.7. How KCs and Macrophages Are Implicated in the Different Stages of MASLD

#### 3.7.1. Gut–Liver Axis and KCs

A correlation of the intestinal microbiota in the feces from biopsy-proven MASH patients and healthy controls with the hematopoietic cell marker CD45 and the KC marker CD163 demonstrated that the abundance of *Faecalibacterium prausnitzii* was negatively correlated with CD45+ and CD163+ cells in the portal tracts [399]. This indicated a possible relationship between the intestinal microbiota and KC activation in analogy with autoimmune hepatitis patients with increased intestinal permeability and pronounced RIP3-mediated activation of liver KCs [400]. As mentioned before, the LPS-TLR interaction is important for the communication between the intestinal tract and liver in MASH [393]. Interestingly, it was shown that the gut produces HDL3, which is a distinctive high-density lipoprotein associated with a specific defense mechanism. It forms a complex combined with LPS and LPS-binding protein (LBP) in portal blood that prevents the binding of LPS to TLR4 in KCs and significantly represses the inflammatory response of KCs [401].

Bile acids also connect the gut with KCs through the TRG5 receptor. TGR5 expression was significantly reduced in the liver tissue of MASH patients and murine models. Deletion of TGR5 promoted KC M1 polarization in mice and increased inflammation, indicating that TGR5 signaling mitigates macrophage M1 polarization, hepatic steatosis, and inflammation [402].

Finally, an additional link between intestinal dysbiosis and MASLD was suggested when intestinal lamina propria macrophages expressing the fractalkine receptor CX3CR1 were shown to be crucial in preserving intestinal barrier integrity [403]. Impairment of the intestinal barrier and severe steatohepatitis were observed in Cx3cr1-deficient mice [404].

On the other hand, activation of FXR in macrophages leads to a switch toward the anti-inflammatory M2 phenotype, increasing IL-10 production, and decreasing the production of IL-6 and INF-γ [158].

#### 3.7.2. KCs and Insulin Resistance

The mechanism of increased hepatic steatosis in IR is unclear and remains controversial. In the normal liver, insulin increases glucose consumption and de novo lipogenesis. In MAFLD, insulin-resistant individuals have unrestrained hepatic glucose production but paradoxically also have increased liver lipid synthesis, leading to hyperglycemia and hypertriglyceridemia [405,406]. Insulin resistance appears in obese individuals. Excess fatty acids, due to diet habits and increased lipogenesis, accumulate in the liver and adipose tissue [407]. Transplantation of visceral adipose tissue from obese mice increased liver macrophages and liver injury, compared to mice that received transplants from lean donors. Adipose tissue macrophage depletion prior to transplantation mitigated this effect [408], indicating that a collaboration between the liver and adipose tissue exists.

These findings are in agreement with recent studies demonstrating that the accumulation of microbial DNA in tissues other than the liver is associated with obesity and contributes to the development of insulin resistance [409,410,411]. EmKCs are protectors against microbial metabolites by expressing the complement receptor of the immunoglobulin superfamily (CRIg), which is responsible for the phagocytosis of pathogens [246]. Obesity reduces the CRIg+ KC population, leading to the accumulation of microbial DNA in the liver and adipose tissue [412]. Overexpression of CRIg in KCs of obese mice protects against bacterial DNA accumulation in metabolic tissues. Mechanistically, the serine/arginine-rich splicing factor 3 (SRSF3) regulates CRIg expression, which is essential for maintaining the CRIg+ KC population. During obesity, SRSF3 expression decreases, followed by a reduction in the CRIg+ population, which is restored with weight loss. Restoring SRSF3 in KCs attenuates obesity-related insulin resistance [413].

Obesity also causes a shift in the polarization of emKCs towards a pro-inflammatory phenotype, which is also a contributing factor to insulin resistance and tissue inflammation [414,415]. Depletion of KCs can protect against insulin resistance and liver steatosis [416].

Using a macrophage-specific proliferation inhibition mouse model, it was demonstrated that macrophages in adipose tissue, liver, and skeletal muscle were necessary for the development of systemic insulin resistance [417].

The influence of vitamin D in insulin resistance has also been investigated. KCs express the highest level of vitamin D receptors (VDRs) compared to other sinusoidal cells, while VDR expression is very low in hepatocytes. VDR activation by the vitamin D analog calcipotriol was reported to attenuate hepatic steatosis, improving insulin resistance. Ablation of KCs eliminated the beneficial effects of calcipotriol [418].

An additional indication of the significance of KCs in the development of insulin resistance was provided by a report demonstrating that KC-specific cannabinoid-receptor type 1 deficient obese mice had improved glucose tolerance and insulin resistance [419].

Furthermore, p38a expression is increased in biopsies of patients with MASLD. Deletion of p38a from mice macrophages led to M2 polarization and improved insulin resistance in murine models of MASLD [420,421], indicating that insulin resistance and progression of MASLD are p38a-dependent.

Most recently, mice deficient in G-protein coupled receptor 3 (GPR3) were shown to develop late-onset obesity [422,423], suggesting a role of GPR3 in regulating metabolism.

In a murine model, activation of GPR3 in KCs increased glycolysis and protected mice from obesity and fatty deposition in hepatocytes, being an additional indication that KCs are implicated in the development of insulin resistance. In liver biopsies from MASLD patients, GPR3 activation increased expression of glycolytic genes and reduced expression of inflammatory genes in KCs [424].

Finally, a very recent report corroborated the involvement of KCs in the regulation of insulin resistance. GABA administration attenuated HFD-induced hepatic insulin resistance by repressing TLR4/NF-κB activation in KCs, leading to reduced secretion of inflammatory cytokines [425].

#### 3.7.3. Initial Stages of MASLD-MASH

KCs are the first responders to lipotoxicity, oxidative stress, and PAMPs in early MASLD. These stimuli activate PRRs [426,427,428]. KCs also respond to FFAs, LPS, and DAMPs in MASLD [429]. Activated KCs secrete pro-inflammatory cytokines contributing to the recruitment of additional immune cells such as T lymphocytes and neutrophils [79,430]. M1 polarization of KCs in MASLD maintains inflammation and inhibits repair mechanisms induced by M2-polarized macrophages [431,432].

Earlier studies in murine models have demonstrated cholesterol accumulation in the lysosomes of KCs in the early stages of MASLD through LDLR-mediated endocytosis or the scavenger receptor CD36, which binds oxLDL particles [321,433,434]. Experiments with deletion of scavenger receptors reduced lysosomal cholesterol accumulation and ameliorated inflammation in MASH [317,435,436,437]. At the early stages of MASH development, the emKC pool is gradually replaced by BMDMs, as already mentioned. An argument was put forward that, in several reports, the reduction in KCs has been quantified as a proportion of the total number and not as an absolute number. However, in MASLD, such quantification has been performed and a decline in KCs was ascertained by whatever marks were used for identification of KCs [279,280,295,302]. However, loss of emKCs and the replacement by infiltrating BMDMs is an event that characterizes all inflammatory injuries of the liver, including MASH-associated HCC and experimental diethylnitrosamine-induced HCC [438,439]. During recovery, the number of emKCs was normalized, and the level of recruited BMDMs returned to the baseline [303].

Earlier research has shown that the deletion of hepatic macrophages prevented the development of insulin resistance and liver hepatic deposition in diet-induced murine models of MASLD [440,441]. This was attributed to the silencing of the macrophage Nf-kb gene and the subsequent reduced production by macrophages of IL-1β [442]. This prevailing idea was challenged by a more recent report, where specific repression of macrophage IL-1β did not improve insulin resistance in obese mice, indicating that it is NF-kB and not IL-1β that modulates insulin resistance, at least in the HFD-induced model. However, more than 1000 genes were altered by insulin resistance in the same model [443]. The majority of these genes in both mice and humans are implicated in the oxidative stress developed as a result of abnormal lipid peroxidation and excessive production of ROS [97,444]. Macrophage MiR-204-3p has also been identified as a crucial regulator of MASH. Reduced miR-204-3p levels in macrophages promoted inflammation and fat deposition and were correlated with disease severity. MiR-204-3p inhibited the pro-inflammatory TLR4/JNK signaling pathway and promoted autophagy [445]. A very interesting recent paper suggested a new approach to the early development of MASH. A model of maternal obesity was used to demonstrate that fetal KC function was disturbed during gestation. Offspring developed fatty liver, which was maintained into adulthood due to increased lipid uptake by hepatocytes. Moreover, KCs deletion in these mice and restitution by normal BMDMs abrogated this effect. Furthermore, ablation of the HIF1-a gene also prevented KC genes from being in macrophages, which prevented the development of MASLD [446].

A few studies have identified liver macrophages with anti-inflammatory phenotypes maintained by the fatty acid sensor peroxisome proliferator-activated receptor-δ (PPARδ) [415]. Moreover, these anti-inflammatory M2 liver macrophages induced apoptosis of pro-inflammatory M1 macrophages via caspase 3, thereby preventing their pathological effects during MASLD [369]. Taken together, these findings demonstrate the role of macrophages as both initiators and perpetuators of liver inflammation.

#### 3.7.4. Progression of Inflammation in MASLD/MASH

Macrophages are important for the progression from steatosis to MASH, being the central mediators of sustained inflammation and fibrogenesis [98]. Inflammatory mechanisms operate from the early stages of MASLD, but they are particularly active in advanced disease stages, including cirrhosis or during development of HCC [68,447]. Early clinical studies indicated that macrophages were involved in the progression of MASLD. A study on young Koreans showed a high number of CD68 + KCs in biopsy tissues from patients with advanced MASLD [448]. In another report, high numbers of activated macrophages were localized among injured hepatocytes in children with MASLD [449]. Although the specific roles of KCs and BMDMs are difficult to distinguish, it seems that both populations are involved in hepatic inflammation and fibrosis through the secretion of pro-inflammatory cytokines and TGFβ1 [28]. The newly recruited BMDMs are immature and more inflammatory compared with emKCs, promoting both hepatocyte damage and fibrosis during MSLD progression. The reason for this different behavior has not yet been elucidated [450,451,452]. Prolonged administration of a high-fat-high-sugar diet in mice resulted in protracted exposure of KCs to DAMPs, leading to protracted inflammasome activation and macrophage-driven fibrosis and HCC [453]. In prolonged hypernutrition, the continuous production of pro-inflammatory cytokines such as TNFα and IL-1β increased proteolytic degradation of the macrophage phagocytic receptor trem2, which is necessary for efferocytosis of lipid-laden apoptotic hepatocytes and induction of LAMs (FIG 2). A reduction in trem2 led to an abnormal accumulation of damaged hepatocytes, further promoting pro-inflammatory cytokine production and establishing a vicious circle that facilitates the progression to MASH [350]. This observation could well be the turning point for the transition from MASLD to MASH.

Activated M2 macrophages produce high levels of TGF-β1 and IL-13, leading to the development of fibrosis [454]. Interestingly, KCs can secrete and respond to both pro-inflammatory cytokines, such as IL-6, and anti-inflammatory cytokines, such as IL-10. In addition, IL-10 production by macrophages promoted and mitigated hepatic inflammation [455].

Another potential mechanism that may potentiate inflammation and fibrosis in MASH is activation of YES-associated protein (YAP). LPS was found to activate YAP localized in KCs, upregulating the secretion of pro-inflammatory cytokines. This effect was abrogated in TLR4-deficient macrophages [456]. In another experimental model, activation of YAP was obtained by deletion of the mammalian Sterile 20-like kinase 1 and 2 (Mst1/2). YAP activation resulted in upregulation of both inflammatory and reparative functions of KC1 and KC2, increasing inflammation and fibrosis. Moreover, YAP activation prolonged the lifespan of KC2 cells but not of KC1 cells [457].

A consequence of fibrosis and the abnormal perfusion of the sinusoids was a change in KC identity markers (CLEC4F, CRIg, and TIM-4) resulting in a reduction in their phagocytic capability and clearance of bacteria. As an adaptation process, phagocytic Kupffer cell-like macrophages forming multinucleated syncytia were described in deranged sinusoids, which were possibly the result of fusion of several infiltrating BMDMs. Whether this is also true in MASLD-associated fibrosis remains to be established [458,459]. The interaction of KCs and BMDMs with natural killer T (NKT) cells aggravates liver injury in murine models of MASLD, intensifying inflammatory pathways [89,460,461].

A series of recent reports indicated, in animal models of MASLD, that additional factors may be implicated in MASH progression. Evidence has indicated that the IQ Motif Containing GTPase Activating Protein 1 (IQGAP-1) may be important in the progression of MASLD. In a murine model of MASLD, increased expression of IQGAP-1 was found in KCs. A lack of IQGAP-1 significantly reduced the levels of TGF-β within KCs. Moreover, incubation of HSCs with macrophage-conditioned medium from IQGAP-1-deficient cells markedly inhibited HSC activation, indicating that IQGAP-1 regulates the KCs-HSCs axis in advanced MASLD [462]. Galectin-12 may also promote fibrosis development in MASH. Deletion of galectin-12 increased M2 polarization of KCs. M2-type KCs mitigated hepatic inflammation by repressing the activation of M1-type KCs, but they promoted fibrosis [463]. However, a different result was reported on the role of M2 polarization and fibrosis. Metalloproteinase 10 (MMP-10) produced in response to M2 polarization mitigated MASLD progression and fibrosis. During MASLD progression, IL-4 promoted PPARγ translocation to the nucleus of macrophages to increase MMP-10 expression. MMP-10 in turn promoted the M2 polarization of macrophages which inhibits the release of pro-inflammatory cytokines and increases the release of the anti-inflammatory cytokine IL-10, leading to amelioration of MASLD progression [464]. Recent reviews support the suggestion that macrophages promote liver fibrosis through the secretion of IL-1β and TNF-α by M1 macrophages, while M2 macrophages ameliorate liver fibrosis [118,465,466].

Another discrepancy concerning the participation of macrophages in fibrosis and the progression of MASH is the characteristic existence of hepatic crown-like structure (hCLS) in MASH. The origin of macrophages participating in hCLC structures is disputed. It was mentioned above that LAMs originating from recruited BMDMs are the macrophages of hCLS. On the contrary, it was reported that CD11c+ macrophages surround damaged hepatocytes and form hCLS. Moreover, it was demonstrated that KCs were a major cellular source of these CD11c+ macrophages. Depletion of CD11c+ macrophages abrogated hCLS formation and fibrogenesis in MASH [467]. In a recent paper, it was confirmed that CD11+ macrophages formed the HCLS structures. However, it was suggested that they originated from recruited macrophages and not from resident KCs. In addition, a co-localization of CD11+ cells with collagen fibers was described, indicating that these cells may promote fibrosis. By contrast, CD11− cells may promote tissue repair [468].

These discrepancies possibly reflect the inability of the current markers to identify the various subsets of liver macrophages. The plasticity and transformation of one subset of macrophages into another, and the fact that, in different experimental models, this transition varies, may be an additional explanation for these discrepancies. The influence of phenotypically heterogeneous macrophage subsets is demonstrated by a clinical study showing that increased levels of osteopontin, monocyte chemoattractant protein (CCL2), and IL8 in serum were associated with upregulation of their genes in liver biopsies of patients with MASH advanced fibrosis [355]. However, the current suggestion is that BMDMs, rather than KCs, are the producers of factors required for fibrosis progression in MASLD [284,469].

#### 3.7.5. Macrophage Exosomes and Fibrosis

Exosomes are extracellular lipid bilayer vehicles (EVs) ranging in size from 30 to 150 nm which facilitate communications between cells. Exosomes may promote or inhibit liver fibrosis as well as macrophage polarization by transporting miRs [470,471,472]. Exosomes derived from the macrophage cell line THP-1 after incubation with LPS contained miR-103-3p, leading to HSC activation and proliferation [473]. In addition, LPS stimulation of THP-1 cells produced exosomes rich in miR-155-5p, which promoted oxidative stress and collagen synthesis by HSCs [474]. Moreover, LPS-induced macrophage activation and production of exosomes rich in miR-500 led to HSC activation [475]. On the other hand, exosomes rich in miR-411-5p derived from M2 macrophages inhibited HSC activation and downregulated collagen and α-smooth muscle actin (α-SMA) [476]. Exosomes produced by lipotoxic hepatocytes rich in miR-192-5p activated M1 macrophage polarization in a murine model of MASLD [477]. M1 macrophage polarization was also promoted by exosomes from dying hepatocytes overexpressing lipid-induced death receptor 5 on their surface [478]. Lysosomal stress caused by cholesterol overload mediated production of hepatocytic exosomes rich in miR-122-5p, which induced M1 polarization [479]. Furthermore, exosomes originating from activated HSCs stimulated pro-inflammatory polarization of macrophages [480]. In contrast, miR-148a in exosomes from mesenchymal stem cells changed macrophage phenotype, switching it from M1 to M2 subsets [481]. In summary, current evidence indicates that exosomes from various sources and different contents of miRs are heavily involved in liver inflammation and fibrosis, acting either directly on HSC or indirectly through pro-inflammatory polarization of macrophages [482].

### 3.8. Macrophages and Adipocentric Theory

Macrophages are critical pillars in the adipocentric theory of MASLD. A link between dysregulated metabolism in the adipose tissue and activation of liver macrophages was reported in patients with MASLD. Interestingly, the association was not dependent on the presence of obesity or diabetes and was attributed to the increased flow of FFAs [483]. An additional explanation for the interplay between adipose tissue macrophages (ATMs) and the liver was provided in a very recent paper. ATMs produced EVs rich in pro-fibrogenic miR-155 and miR-34a. In vitro EVs from MASH-associated ATMs induced HSC activation and exacerbated liver fibrosis when injected into obese mice [484]. The secretion of adipokines is responsible for most aspects of the interplay between adipose tissue and hepatic macrophages. Adiponectin can direct KCs and macrophages toward an anti-inflammatory phenotype [485] and also reduced the proliferation and migration of HSCs and KCs, repressing fibrosis [486,487]. Deletion of adiponectin promoted fibrosis in a murine model [488]. Interestingly, MASH in turn caused dysfunction of ATMs, inducing a continuous vicious cycle between adipose tissue inflammation and MASLD [489].

Leptin, the other important adipokine, is not produced in the normal liver, but it is produced by activated HSCs during the progression of the fibrotic process [490]. Leptin is also produced by adipose tissue and activates KCs through the leptin receptor (LEPR) [322]. After activation by leptin, KCs produce TGF-b1, which further activates HSCs, inducing another vicious circle to promote fibrosis [491,492,493,494].

There are additional adipose tissue-derived factors with a newly described role in MASLD. WAT of MASLD mice increased the secretion of the protein acidic and rich cysteine-like protein 1 (Sparcl1). Plasma Sparcl1 levels increased in MASLD patients and were positively associated with liver damage. Overexpression of Sparcl1 promoted liver inflammation while deleting Sparcl1 in WAT ameliorated diet-induced MASH. Based on experiments with hepatocyte cell lines, these effects were attributed to activation of TLR4 in hepatocytes, resulting in the upregulation of CCL2, followed by the recruitment of BMDMs. However, the vast majority of liver TLR4 is found in the KCs as mentioned above. Therefore, it is possible that the in vivo findings could be due to Sparcl1’s effect on KCs [495].

Maresins were described as macrophage mediators that promote the resolution of inflammation [496]. Brown adipose tissue-derived maresin 2 (MaR2) is implicated in the repression of hepatic inflammation [497] by decreasing the expression of pro-inflammatory genes in KCs and their secretion after LPS activation. Moreover, MaR2 secreted from brown adipocytes promotes the recruitment of trem2-positive macrophages that initiate a protective, anti-inflammatory response similar to that of LAM cells in MASH as mentioned before [356,498,499].

### 3.9. Key Cellular Interactions Between Liver Macrophages and Other Liver Cell Types in MASLD/MASH

From the evidence presented so far, it is clear that initiation of inflammation in MASLD has several origins, which are both hepatic, such as lipotoxicity or oxidative stress, and extrahepatic, such as the gut–liver axis, adipose tissue, and skeletal muscles, resulting in a complex immune-mediated pathophysiology. In fact, communications among the different liver cells are necessary for the initiation and progression of the disease [74].

In murine models KCs regulate reductions in natural killer T cells via IL-12 [310] and triglyceride accumulation in hepatocytes [478]. BMDMs can either induce hepatic stellate cell activation and proliferation via TGFβ1 [500], TNFα, and IL-1β [501] or may have anti-fibrotic functions by secreting matrix-degrading metalloproteases [502]. In addition, macrophages accept signals sent by other liver cells. During the early stages of MASLD, LSECs initiate recruitment and activation of BMDMs via reduced secretion of NO [503,504]. At later stages, LSECs initiate recruitment of monocytes by upregulating adhesion molecules such as VLA-4 [304,505]. In the later stages of MASLD, hepatocytes induce recruitment and activation of BMDMs via production of EVs, which are absorbed by macrophages [477,478].

Another interplay between hepatocytes and KCs was suggested when a considerable reduction in the level of alpha-1-antitrypsin (A1AT) secreted by hepatocytes was observed in murine models and patients with MASH. Deletion of Serpina1, the gene encoding A1AT, aggravated MASH through increased activity of the pro-inflammatory proteinase 3 (PR3) produced by moKCs. Elevated hepatic IL-1β levels produced by KCs in MASH also downregulated A1AT, resulting in increased recruitment of MoKCs and enhanced PR3 activity [506].

IL-32 has been described as an important cytokine associated with MASH. IL-32 was closely associated with insulin resistance and aminotransferase levels, indicating that IL-32 is implicated in the pathogenesis of MASLD [507]. IL-32 was the most prominent upregulated gene in the fibrotic MASLD, independently of the presence or absence of the PNPLA3 genetic risk variant. Moreover, circulating IL3-2 levels were associated with MASLD independently of the severity of disease or the level of aminotransferases [508]. In contrast to previous studies, overexpression of IL-32γ ameliorated MASH in a murine model by inhibiting inflammation and by preventing HSCs activation induced by KCs. This discrepancy can be explained by the decreased levels of A1AT found in MASH, as reductions in A1AT increased PR3 activity, eliminating the protective effect of IL-32γ. PR3 cleaves IL-32γ, turning it from a protective cytokine into a strong activator of KCs, leading to HSC activation. This process increases liver inflammation and fibrosis [506]. Nuclear receptors (NRs) are also implicated in the communication between liver cells involved in MASLD. Multiple NRs such as farnesoid X receptor (FXR), liver X receptor (LXR), and retinoid X receptor (RXR) are found in both immune and parenchymal liver cells modulating lipid metabolism. In MASLD, lipid lipid-loaded hepatocytes secrete cytokines such as TGFβ and ROS to modulate KC polarization, further promoted by FFA and bile acids. In cooperation with hepatocytes, activated KCs initiate the transformation of HSCs into myofibroblasts to induce fibrogenesis [390].

### 3.10. Macrophage Autophagy and Programmed Cell Death in MASLD

Liver macrophages die through similar mechanisms to hepatocytes, as analyzed in the second section of this review. Programmed cell death (PCD) is involved in the polarization of macrophages. M1 macrophages may survive inflammation, as they are more resistant to ferroptosis in comparison to M2 macrophages [509].

#### 3.10.1. Autophagy

Autophagy of macrophages is dysregulated in human livers with MASLD or MASH. Deletion of autophagy-related proteins such as autophagy protein 5 (ATG5) in macrophages increased IL1α and IL1β secretion, aggravating liver inflammation in mice [510]. Moreover, upregulation of autophagy mitigated the pro-inflammatory activation of human macrophage cell lines [511]. Increased levels of ROS activated the oxidative stress sensor nuclear Nrf2, leading to activation of anti-oxidant genes and induction of selective autophagy in macrophages, thus ameliorating liver injury [512]. Nrf2 was reduced in the livers of MASH patients [444]. Deletion of Nrf2 in macrophages increased ROS and IL-1β production, aggravating MASH progression, while overexpression of Nrf2 in liver macrophages ameliorated MASH [513]. In contrast, a reduction in autophagy in liver macrophages aggravated liver inflammation and fibrosis by enhancing the mitochondrial ROS/NF-kB/IL-1α/β pathway [514]. AMP-activated protein kinase (AMPK) increased macrophage M2 polarization. FFA and hyperglycemia can lead to autophagy deficiency through the inhibition of AMPK, inhibiting macrophage M2 polarization and increasing M1 polarization [515,516,517,518]. Upregulated expression of DNA methyltransferase (DNMT1) reduced autophagy and increased hypermethylation at the promotor regions of autophagy genes such as ATG-5 and ATG-7 in a murine model of MASLD. Inhibition of DNMT1 restored KC autophagy and M1/M2 polarization, preventing the progression of MASLD [519]. Macrophage deficiency of mTORC1, which is an important regulator of autophagy, also inhibited their M2 polarization by inducing lysosomal dysfunction [520]. Most importantly, macrophage autophagy downregulated programmed death ligand 1 (PDL1) expression, inhibiting HCC development, while dysregulated autophagy induced immunosuppression, promoting HCC progression [521].

#### 3.10.2. Apoptosis

Apoptosis is a form of programmed cell death without the liberation of cellular elements into the cell microenvironment. Apoptosis is regulated by a series of activated caspases. An earlier report found increased positive cells after the application of the terminal deoxynucleotidyl transferase-mediated dUTP nick end labeling (TUNEL) assay in the liver tissue of MASH patients, indicating that apoptosis was implicated in the progression of MASH [522]. Caspases participating in apoptosis are classified into two categories: initiating caspases (2, 8, 9, and 10) and effector caspases (3, 6, and 7) [523]. Caspases-3, -6, -7, -8, and -9 promote the progression of MASH [328].

The suppressor of cytokine signaling (SOCS) family is a class of proteins that negatively regulate cytokine signals [524]. SOCS2 expression in macrophages was negatively correlated with the severity of MASH, repressing inflammation and apoptosis of hepatocytes via NF-kB and inflammasome activation in macrophages during MASH [525]. No specific data concerning KCs and BMDMs apoptosis during MASLD progression have been reported.

#### 3.10.3. Necroptosis

Necroptosis is a programmed cellular death process that is not dependent on caspases. It is regulated by receptor-interacting serine/threonine kinase 1 (RIPK1) and RIPK3. Necroptosis favored M1 polarization and reduced M2 polarization of macrophages [526]. Patients with MASH and murine MASLD models showed that hepatic RIPK3 expression correlated with MASLD severity, while RIPK3 deletion alleviated inflammation, fibrosis, and HCC development in mice [527]. However, it was demonstrated in a murine model that RIPK3 deficiency mitigated inflammation but aggravated hepatic fat deposition, indicating that inhibition of necroptosis may promote either increased lipid uptake or increased de novo synthesis of fatty acids [528].

#### 3.10.4. Pyroptosis

The gasdermin E-N domain produced from Gasdermin E (GSDME) by caspase-3 mediated pyroptosis by forming pores in plasma membranes. It also induced mitochondrial ROS release by targeting the mitochondrial membrane and promoting MASH progression [529,530]. Universal deletion of Gasdermin ameliorated liver steatosis, steatohepatitis, fibrosis, ER stress, lipotoxicity, and mitochondrial dysfunction in murine MASH models. Specific overexpression of GSDME in myeloid cells, but not in hepatocytes, restored MASH pathology, including alterations in hepatic macrophage subsets [531]. Inhibition of macrophage pyroptosis may alleviate MASH through NLRP3 inflammasome inhibition [532]. Other inflammasomes such as the NLR family CARD domain containing 4 (NLRC4) are also involved in MASH progression by increasing macrophage pyroptosis [533]. Deletion of myeloid Trem2 aggravated liver fat deposition and inflammation in a model of MASLD, while overexpression of Trem2 had the opposite effect. Deletion of Trem2 increased macrophage pyroptosis and amplified the resulting inflammatory response. In contrast the presence of Trem2 promoted resolution of inflammation, transforming macrophages through TGFβ1 toward M2 polarization [534].

#### 3.10.5. Iron and Ferroptosis

There is a close association between disease progression and hepatic iron overload in MASLD patients [535]. An earlier large study of patients with MASLD demonstrated that iron overload of macrophages is associated with advanced liver histology [211]. Increased ferritin is observed in approximately 30% of patients with MASLD and dysmetabolic iron overload syndrome (DIOS) is found in 15% of patients with metabolic syndrome and in 50% of those with MASLD [536]. Moreover, enhanced expression of the divalent metal transporter 1 (DMT1) was described in patients with MASLD, leading to increased absorption of iron from the GI tract [537]. The iron status in the liver of patients was associated with the intestinal microbiome. In particular, there was a positive association with *Bacteroides* and *Prevotella* spp. [538]. In a murine experiment, treatment with iron of BMDMs resulted in upregulation of M1 markers, an effect that was reversed by an iron chelator. Iron loading of macrophages in the presence of IL-4 downregulated M2 markers. MASLD patients with iron deposition in liver macrophages had increased hepatic levels of M1 markers and decreased levels of one M2 marker [539]. Interestingly, it was reported that the characteristic crown-like structures observed in MASH are surrounded by iron-rich, CD11c-positive KCs, as discussed before. Iron overload in these cells led to lysosomal stress, which in turn activated the transcription factors MiT/TFE in CD11c-positive KCs. The end result was the formation of hCLS and the aggravation of inflammation and fibrosis [540]. There is evidence that KCs and other macrophages modulate ferroptosis by secreting inflammatory cytokines. IL-6 secretion from polarized M1 KCs initiated ferroptosis by inducing ROS-dependent lipid peroxidation [541] and by depleting antioxidants, such as GSH and glutathione peroxidase 4 (GPX4) [542]. TNF-α produced by M1 macrophages induced acyl-CoA synthetase 3, promoting the production of lipid droplets and ferroptosis [543,544]. The proteoglycan decorin (DCN) is liberated by lysosomal exocytosis during ferroptosis and initiates immune responses. Extracellular DCN binds to the receptor advanced glycosylation end-product-specific receptor (AGER) on macrophages to initiate the production of pro-inflammatory cytokines in an NF-κB-dependent manner [545]. The participation of these two pathways in MASLD has not been investigated.

### 3.11. HCC in MASLD

HCC may appear early in MAFLD without cirrhosis. Early diagnosis is based on dynamic imaging tests such as MRI and liver ultrasound with enhancement by contrast agents. Ultrasound-guided liver biopsy remains the gold standard in diagnosis, which is also the gold standard for fibrosis assessment [546].

The hepatic microenvironment during progression of MAFLD supports the development of malignant transformation of hepatocytes and initiation of HCC [547]. KCs and BMDMs are responsible for immune surveillance as they are involved in the phagocytosis and elimination of premalignant hepatocytes at the early initiation of HCC [548], but this monitoring can be bypassed [549]. BMDMs infiltrating HCC may develop into tumor-associated macrophages (TAMs) [547] characterized by the expression of Trem2, GPNMB, C1QA, and C1QB resembling not only immune-repressive, tumor-permissive LAMs [356,550,551] but also TAMs associated with other types of cancer [552,553]. Liver macrophages are implicated in practically all MASLD- and HCC-related signaling pathways as orchestrators of disease progression with either beneficial or detrimental functions [554].

Obesity-promoted HCC initiation is associated with enhanced levels of IL-6 and TNF-α [555]. In a mouse model of ER stress and lipid deposition, M1-derived TNFα not only initiated HCC development [556] but also promoted malignant cell survival and proliferation via NF-κB activation [557]. Cell clearance by efferocytosis has drawn attention in MAFLD as it can indirectly promote HCC development through promotion of inflammation and fibrosis. Thus, prolonged hypernutrition in fatty livers impairs Trem2-dependent macrophage efferocytosis, aggravating inflammation and MASH progression [350]. The accumulation of necroptotic, but not of apoptotic, hepatocytes in human and mouse MASH livers is controlled by the upregulated “don’t-eat-me” CD47 molecule on necrotic hepatocytes. The binding of CD47 to its receptor, SIRPα, on liver macrophages inhibits the clearance of necrotic hepatocytes, leading to increased HSC activation and progression of fibrosis [558]. Macrophages’ phagocytic capability is controlled by the balance between “eat me” and “do not eat me” ligands on cells that are guarded by patrolling macrophages. “Do not eat me” ligands, like CD47 and PDL1, are frequently upregulated in tumors and protect the malignant cells from phagocytic elimination [559].

Aberrant signaling from immature macrophages could enhance tumorigenicity in MASLD, but little is known about the metabolic alterations of the KCs/BMDMs, which may determine the fate of both the macrophages and HCC progression. Metabolic stress may impair cytotoxic effector immune cells, promoting immune evasion in HCC [560]. Macrophage polarization depends on cellular metabolic pathways. Increased glycolysis and fatty acid synthesis and increased function of the pentose phosphate pathway coupled to decreased oxidative phosphorylation (OXPHOS) activity are characteristics of M1 macrophages favoring ROS production [396,561]. By contrast, M2 macrophages have increased OXPHOS and fatty acid oxidation with reduced glycolysis, promoting immunosuppression [561,562]. TAMs often switch between the two phenotypes [554,563]. In early HCC, TAMs use glycolysis, but later on switch to OXPHOS-dependent metabolism [564]. Lactate, the principal product of aerobic glycolysis, is the central mediator for this switch [565]. Lactate production by HCC cells activates HIF-1α, increasing the expression of pro-tumorigenic genes [566]. Enhanced glycolysis in TAMs is associated with elevated PD-L1 expression [567]. Elevated lactate levels upregulate IL-23, promoting a pro-inflammatory but immunosuppressive environment [568]. Lactate also modifies the activity of the nuclear factor of the activated T cells (NFAT) in both T and NK cells, diminishing antitumor defenses by reducing IFNγ secretion [569,570].

An additional factor involved in HCC development in MASH is fibroblast growth factor 21 (FGF21). FGF21 is mainly produced in the liver [571], and protects the liver from the metabolic stress induced by MASH [572] through a multitude of endocrine functions, including, among others, control of lipolysis and clearance of surplus of FFAs that negatively regulate fat deposition [573,574]. It inhibits TLR4–interleukin-17A signaling, preventing the MASH-HCC transition [575]. It should be noted that TLR4 in meKCs has an important role in the development of HCC in MASLD [576,577]. A lack of FG21 provides increased amounts of FFAs in the sphingosine-1-phosphate (S1P) cascade to mediate the MASH-HCC transition via the S1P-YAP pathway and interplay between HCC cells and macrophages [573].

Amino acid metabolism is also implicated in the development of the tumor microenvironment in HCC [578]. Upregulation of asparagine metabolism is accompanied by immunosuppression characterized by upregulation of Alanine/Serine/Cysteine Transporter 2 (ASCT2) and downregulation of Glutaminase 2 (GLS2) expression [579,580]. This alteration is followed by an increased infiltration of regulatory T cells and M2 macrophages and a reduction in M1 macrophages and cytotoxic effector T cells, promoting HCC progression [581]. Whether these changes occur in MASLD-associated HCC is not yet clear.

In summary, the HCC liver microenvironment is dominated by a variety of immune cells, where TAMs derived from either emKCs or BMDMs have a dominant role. Cells with anti-tumor activity, such as NK cells, CD8+ cytotoxic T cells, and KCs, are defective. NK cells are downregulated and suppressed by Tregs and Myeloid-derived suppressor cells (MDSCs), which are a heterogeneous group of immune cells that originate from bone marrow stem cells. KCs may produce IL-6 to promote tumor development, while their anti-tumor activity is repressed by MDSCs. TAMs favor neo-angiogenesis and promote HCC progression by producing IL-6 and TGFβ and by inhibiting NK cells in collaboration with MDSCs. They also recruit and activate T regs [582].

### 3.12. Treatment of MASLD by Targeting Macrophages

Several effective methods to target liver macrophages in the treatment of MASH have been reviewed that may be clinically tested or could be tried in the future [583]. However, it should be stressed that most drugs currently tested for treatment of MASLD may also partially act through macrophage manipulation. FXR agonists reduce the production of pro-inflammatory cytokines from macrophages and switch the polarization of macrophages toward an M2 phenotype [584,585]. Animal studies indicated that FXR agonists decrease the production of pro-inflammatory cytokines by KCs, attenuating liver inflammation induced by LPS stimulation of KCs [586]. These findings suggest that the beneficial effects of FXR activators in MASH may be partially due to their effect on KCs. Clinical trials demonstrated the effectiveness of obeticholic acid, an FXR agonist, in inhibiting hepatic glucose and lipid metabolism, in addition to its anti-inflammatory and anti-fibrotic properties in MASLD [587].

Macrophage metabolism is also a target of MASLD treatment [322]. The use of glucagon-like peptide-1 receptor agonists (GLP1RAs) have been tested for the treatment of MASLD [588,589,590]. In a clinical study, liraglutide showed partial histological improvement in MASH [591]. Furthermore, Gliptins, dipeptidyl peptidase 4 inhibitors, which indirectly activate GLP1R, were shown to decrease the number of M1 macrophages in the liver and switch macrophage polarization toward the M2 anti-inflammatory phenotype in murine models [592].

The stimulation of peroxisome proliferator-activated receptor gamma (PPARγ) through agonists like pioglitazone promotes the conversion of macrophages into an anti-inflammatory state. This conversion has been shown to alleviate hepatic steatosis by enhancing the uptake and breakdown of fatty acids [315,593]. Pioglitazone treatment repressed the activity of M1 macrophages and simultaneously upregulated the expression of M2 macrophages in mice with deficiencies in PPARγ expression in macrophages [594]. Similarly, peroxisome proliferator-activated receptor delta (PPARδ) regulated the polarization of macrophages toward the M2 phenotype [415]. Elafibranor, a dual agonist for PPARγ and PPARδ, has shown improved effectiveness in treating MASH compared to a placebo without worsening fibrosis progression [595]. Currently, saroglitazar, a PPAR-α/γ agonist, has been approved for sale in India and Mexico [596]. In a murine model of MASH, the administration of cenicriviroc (CVC), a dual antagonist of CCR2 and CCR5, significantly improved fibrosis and inflammation [597]. Another study indicated that cenicriviroc reduced macrophage numbers and fibrosis in mouse models of MASH [469]. Clinical trials demonstrated that twice as many patients showed improved fibrosis without worsened steatohepatitis compared to controls [598]. However, a recent phase III study did not find any efficacy of CVC in MASH-related fibrosis, as assessed by histology [599], possibly because the presence of both CCR2 and CCR5 may lead to inhibition of both the detrimental and beneficial effects of the drug [600].

The first drug to receive approval in both the United States and Europe for non-cirrhotic MASH with mild to advanced fibrosis was resmetirom [238]. Resmetirom downregulated pro-inflammatory cytokines, mitigated KC activation, and reduced fibrogenesis by repressing TGF-β signaling [601]. The increased macrophage infiltration that was observed in the liver of a murine model of MASH was restored back to normal by resmetirom, indicating that its activity in MASH may be partly due to an effect on liver macrophages [602].

Fibroblast growth factor 21 (FGF21) plays a positive and protective role in MASLD, as mentioned above. It alleviates hepatocyte fat deposition and lipotoxicity, ameliorates insulin resistance, reduces oxidative stress, and can be used as a therapeutic modality in MASH [603]. Drugs acting on FGF21 have been recently reviewed [604].

The link between programmed cell death (PCD)-regulated macrophage polarization and lipid metabolism suggests that drugs regulating lipid metabolism may also treat MASLD. Modulation of PCD of macrophages inhibits their M1 polarization. Ezetimibe blocked NLRP3 inflammasome activation in macrophages mediating activation of autophagy [511]. Sodium-dependent glucose transporter 2 (SGLT2) inhibitors control blood glucose by inhibiting the reabsorption of glucose from the proximal tubules of the kidney, but also regulate lipid metabolism [605]. Empagliflozin, an SGLT2 inhibitor, confirmed the significance of the AMPK/mTOR pathway in enhancing the autophagy of liver macrophages in murine models with MASLD [606]. Interestingly, a very recent paper suggested that arctigenin, a monomer of Fructus Arctii, inhibited hepatic inflammation by targeting macrophage NLRP3 inflammasomes and prevented the progression of MASH [607]. Mesenchymal stem cells (MSCs) and MSC-derived EV administration switched the balance toward the M2 phenotype in murine models, substantially ameliorating liver inflammation. The possible harmful effect of fibrogenesis progression due to M2 domination is counteracted by the concomitant inhibition of HSCs and the protection of hepatocytes from apoptotic death. This therapeutic approach is considered to be more advantageous compared with other KC-targeted therapies under development [608]. Finally, the galectin-3 inhibitor belapectin, a candidate new drug for preventing and treating MASH, proved to be safe and well tolerated in phase I clinical trials and is currently undergoing phase III clinical studies [609,610,611]. A detailed review on drug treatment of MAFLD has been recently published [395]. Table 1 summarizes the available medications and their effects on liver macrophages.

In summary, the best available drug treatment at this time is resmetirom and elafibranor until phase III studies and real-world trials for other potential therapies are completed.

## 4. Conclusions

MASLD is the manifestation of metabolic syndrome in the liver. The pathogenesis of the disease is multifactorial and not fully elucidated. There is a bidirectional relationship between the liver and extrahepatic organs. Initiators of inflammation are localized in the liver, including lipotoxicity, iron overload, oxidative stress, and endoplasmic reticulum stress. Factors of inflammation are also extrahepatic, such as microbial products from the gut and toxic lipids coming from skeletal muscles and adipose tissue. At the same time, liver damage induces abnormalities in the extrahepatic organs, establishing a vicious circle. The critical factor for the transition from mild MASLD to active MASH, fibrosis, and possibly HCC is not known. In the center of this cascade is the macrophage, which is both a regulator of inflammation and an executioner. It may well be that macrophages be the key to the serious transition toward advanced stages of MASLD. In particular, Kupffer cells and bone marrow-derived macrophages are the key-holders of practically every aspect of this multifaceted disease. They not only participate in the initiation of the disease but also in the development of fibrosis and HCC. Moreover, most available drugs under trial have a significant effect on the function of macrophages. However, the exact role of either Kupffer cells or marrow-derived macrophages has not been clarified due to their immense plasticity and the lack of proper specific markers. The current markers are not always able to recognize the newly described subsets of macrophages and identify their ontogeny with precision. Progress in this field will provide valuable information in the future.

## Figures and Tables

**Table 1:** Medications proposed for the treatment of MASLD. Effects on liver macrophages.

Group and Names	Mode of Action	References
FXR agonists—Obeticholic acid	Reduction in the production of pro-inflammatory cytokines from macrophages; switch the polarization of macrophages toward an M2 phenotype.	[584,585,586,587]
GLP1 Receptor agonists—Liraglutide, Semaglutide	Downregulation of the expression of inflammatory cytokine genes in adipose tissue by suppressing the NF-kB pathway. GLP-1 analogs improve insulin sensitivity in adipose tissue.	[226,227,228,229,230,588,590,591]
Gliptins—Linagliptin, Saxagliptin, Sitagliptin	Decrease the number of M1 macrophages in the liver and switch macrophage polarization toward the M2 phenotype.	[592]
PPAR γ agonists—Pioglitazone	Promotes the conversion of macrophages into an anti-inflammatory state.	[231,232,233,315,593,594]
PPAR δ agonists—Seladelpar	Regulate the polarization of macrophages toward the M2 phenotype.	[415]
PPAR γ/δ agonists—Elafibranor	Switch macrophages in the ileum to the M2 phenotype, improving intestinal integrity to alleviate MASH.	[595]
PPAR α/γ agonists—Saroglitazar	Interferes with transforming growth factor-β/Smad downstream signaling, reducing liver fibrosis progression.	[596]
CCR2/CCR5 antagonists—Cenicriviroc	Reduced macrophage numbers and fibrosis in mouse models of MASH. No efficacy in MASH fibrosis.	[597,598,599]
THR-β partial agonist—Resmetirom	Downregulated pro-inflammatory cytokines, mitigation of KC activation, and reduction in fibrogenesis by repressing TGF-β signaling.	[601,602]
FGF21—Pegbelfermin		[222,223,603,604]
Ezetimide	Autophagy induction through AMPK activation. Dampening of NLRP3 inflammasome activation in macrophages.	[511]
SGLT2 inhibitors—Empagliflozin	Enhancement of autophagy of liver macrophages in murine models of MASLD.	[606]
Arctigenin	Inhibition of hepatic inflammation by targeting macrophage NLRP3 inflammasomes.	[607]
Galectin 3 inhibitors—Belapectin	Reduction in liver fibrosis and portal hypertension in rats. Negative phase 2b study.	[609,610,611]

## Data Availability

No new data were created or analyzed in this study. Data sharing is not applicable to this article.
